# NEAR trial: A single-arm phase II trial of neoadjuvant apalutamide monotherapy and radical prostatectomy in intermediate- and high-risk prostate cancer

**DOI:** 10.1038/s41391-022-00496-8

**Published:** 2022-01-28

**Authors:** Lui Shiong Lee, Adelene Y. L. Sim, Chee Wee Ong, Xinyan Yang, Cedric C. Y. Ng, Wei Liu, Vikneswari Rajasegaran, Abner M. S. Lim, Edwin Jonathan Aslim, Nye-Thane Ngo, Li-Yan Khor, Ravindran Kanesvaran, John Carson Jr Allen, Kae Jack Tay, John Shyi Peng Yuen, Tsung Wen Chong, Sun Sien Henry Ho, Bin Tean Teh, Melvin L. K. Chua

**Affiliations:** 1 Department of Urology, Sengkang General Hospital, Singapore, Singapore; 2 Duke-NUS Medical School, Singapore, Singapore; 3 Division of Medical Sciences, National Cancer Centre Singapore, Singapore, Singapore; 4 Department of Urology, Singapore General Hospital, Singapore, Singapore; 5 Department of Anatomic Pathology, Singapore General Hospital, Singapore, Singapore; 6 Division of Medical Oncology, National Cancer Centre Singapore, Singapore, Singapore; 7 Center for Quantitative Medicine/Office of Research, Duke-NUS Medical School, Singapore, Singapore; 8 Division of Radiation Oncology, National Cancer Centre Singapore, Singapore, Singapore

**Keywords:** Prostate cancer, Cancer therapy

## Abstract

**Objective:**

Treatment efficacy of androgen deprivation therapy with radical prostatectomy for intermediate- to high-risk prostate cancer is less well-studied. The NEAR trial is a single-arm, phase II investigation of neoadjuvant apalutamide monotherapy and radical prostatectomy (RP) in the treatment of D’Amico intermediate- and high-risk prostate cancer (NCT03124433).

**Materials and methods:**

Patients with histologically-proven, D’Amico intermediate- to high-risk prostate adenocarcinoma received apalutamide 240 mg once-daily for 12 weeks followed by RP + /−lymphadenectomy. Primary outcome was pathological complete response (pCR) rate. Secondary outcomes included rate of biochemical response (defined by PSA < 0.03 ng/mL at week 24 from starting apalutamide without subsequent PSA relapse), treatment-related adverse events, and RP complication rates. Correlative biomarker analyses were performed to examine for molecular predictors of treatment responses.

**Results:**

From 2017 to 2019, 30 patients were recruited, of which 20 and 10 were high and intermediate risk, respectively; 25 completed treatment as per-protocol. We did not observe any pCR on trial; median reduction of cancer burden was 41.7% (IQR: 33.3%–60.0%). 18 out of 25 patients were classified as having a biochemical response (4 did not achieve PSA of <0.03 ng/mL at week 24 and 3 developed PSA relapse subsequently). Dry skin (*N* = 16; 53.3%), fatigue (*N* = 10; 33.3%) and skin rash (*N* = 9; 30.0%) were the most common adverse events, and there was no major peri-operative complication. We observed an association between tumours of low androgen receptor activity and PAM50 basal status with biochemical non-responders, albeit these molecular phenotypes were not associated with pathological response.

**Conclusions:**

A 12-week course of neoadjuvant apalutamide prior to RP did not meet the primary endpoint of pCR in this trial. Tumours with low androgen receptor activity or of the PAM50 basal subtype may have a reduced response to apalutamide.

## Introduction

Radical prostatectomy (RP) or radiotherapy are recommended treatment modalities for localised prostate cancer (PCa) [1]. Treatment recommendation is based on the NCCN or D’Amico risk stratification criteria. While combination radiotherapy and androgen deprivation therapy (ADT) is the standard-of-care for intermediate- to high-risk PCa, the combination of ADT with RP is less established [2, 3].

A previous randomised trial supports the use of adjuvant ADT in pathological node-positive high-risk PCa in prolonging overall survival (OS) [4]. However, the use of neoadjuvant ADT remains debatable. Prospective clinical trials investigating the efficacy of ADT in this setting have failed to demonstrate an impact on OS and biochemical recurrence-free rate, even though pathological responses were observed, leading to higher negative surgical margin rates post-RP [5–8]. To enhance these responses, studies had also looked into the use of neoadjuvant enzalutamide [9], abiraterone acetate [10, 11], and combination enzalutamide and abiraterone acetate [12] with ADT prior to RP. Interestingly, only the triplet combination yielded seemingly higher pathological complete response (pCR) rates [12].

Apalutamide is a third-generation androgen receptor (AR) antagonist, with proven efficacy in non-metastatic castrate-resistant and metastatic castrate-sensitive PCa [13–15]. Given the potency of apalutamide for AR inhibition, we hypothesised that apalutamide could induce a high rate of pCR prior to RP. We therefore conceptualised the NEAR trial (Neoadjuvant Apalutamide and Radical prostatectomy, NCT03124433, ClinicalTrials.gov). Patients with D’Amico-defined intermediate- and high-risk localised PCa were enrolled onto this single-arm, phase II trial investigating the efficacy of a 12-week course of apalutamide prior to RP.

## Materials and methods

### Study participants

This study was approved by the SingHealth Institutional Review Board (protocol no: 2016/2934), and conducted in accordance with the International Conference on Harmonisation guidelines for Good Clinical Practice and the principles of the Declaration of Helsinki. Inclusion criteria were: (1) newly-diagnosed, histologically-confirmed adenocarcinoma of the prostate; (2) age 21 to 75 years; (3) D’Amico intermediate- (cT2b or PSA > 10–20 ng/mL or Gleason’s Score [GS] of 7) or high-risk (cT2c-4 or PSA > 20 ng/mL or GS ≥ 8) PCa who consented for RP; (4) absence of nodal and distant metastasis on staging (magnetic resonance imaging of the pelvis and computed tomography of the body and/or skeletal scintigraphy); (5) no known drug hypersensitivity; and (6) normal liver and thyroid function. Exclusion criteria included: (1) small cell, neuroendocrine, or ductal variants; (2) prior pelvic radiotherapy; (3) history of seizures or psychiatric conditions requiring anti-psychotic therapies; (4) renal impairment and serum creatinine more than twice ULN; (5) history of other malignancies ≤ 5 years to diagnosis; and (6) ECOG performance status of ≥ 2. Patients who received prior ADT were eligible, but were required to have a wash-out period of three months before recruitment. All patients provided written informed consent to trial participation.

### Treatment protocol

Patients received 240 mg apalutamide daily for 12 weeks followed by RP within 6 weeks from the last administered dose, which was based on the 7-day half-life of apalutamide and steady state plasma drug levels after 21 days of continuous administration. All patients who received at least one dose of apalutamide had follow-up visits for assessments of safety. RP was performed via a robotic-assisted approach, and the pelvic node dissection template was left to the discretion of the surgeon. Data on adverse events (AEs) were graded according to the Common Terminology Criteria for Adverse Events (CTCAE) version 4.0. All complications occurring peri-operatively and within 30 days of surgery were recorded and stratified according to the Clavien-Dindo grade classification [16] (see Supplementary Appendix).

### Tumour sampling and transcriptome profiling

All diagnostic biopsies and RP specimens were retrieved for tumour sampling. Following pathological assessment, tumours were delineated and microdissection performed using the ArcturusXT LCM (Applied Biosystems, CA) system. Quality control of tissue sampling was performed using an in-house real-time polymerase chain reaction (RT-PCR) method, which assesses amplifiability on three housekeeping genes (actin beta, glyceraldehyde-3-phosphate dehydrogenase, and succinate dehydrogenase complex flavoprotein subunit A). RNA was amplified using RT-PCR via a targeted RNA approach (Ampliseq for Illumina Transcriptome Human Gene Expression Panel, CA) to study gene expression levels using an input RNA of ~10 ng. Sequencing was conducted using Illumina HiSeq 4000 sequencer (Illumina, CA). Data was processed and analysed using the Illumina Local Run Manager RNA module, from which counts per transcript were obtained and aggregated by summation per gene.

### Statistical considerations

The primary endpoint of this study was pCR at the time of RP after a 12-week course of apalutamide. For the calculation of sample size, we used a baseline rate of 10% based historical data from the neoadjuvant ADT and flutamide trials, which observed pCR rates of 4% to 9% [6, 8]. We assumed that apalutamide would yield an additional 15% pCR [12, 17]. Using a one-sided Fisher’s exact test at an alpha of 0.05, we derived a sample size of 30 patients, which would provide 80% power to reject the null hypothesis H_0_: *π* = 0.10 vs. H_1_: *π* = 0.25, where *π* is the true pCR rate. Secondary endpoints included biochemical and pathological response rates. A biochemical response was defined as a PSA level of <0.03 ng/mL at week 24 of the study. Pathological response was defined as the change in tumour burden post-apalutamide (ΔCB) and tissue response, as described by Efstathiou et al. [18]. The pre-treatment cancer burden (Pre-CB) in the primary tumour was estimated using the maximum core involvement on diagnostic needle biopsy. Residual cancer burden (RCB) was calculated based on pathological assessment of tumour dimensions within the prostate gland (eFigure 1). ΔCB was calculated from the difference between pre-CB and RCB. A pCR would be a ΔCB of −100%. For tissue response, grade A is the most favourable with majority of cancer cells in the tumour appearing as clusters, cords, or in isolation; grade B tumours have intact and fused small glands, while grade C is the least favourable with the presence of cribriform glands and/or intraductal spread of tumour cells. Pathological review of the diagnostic and RP specimens was performed centrally by two pathologists (LK and NN). Biopsies were scored based on the International Society of Urological Pathology (ISUP) grading system and the modified Gleason grading system [19, 20].

Secondary outcomes included the proportion of treatment-related AEs after neoadjuvant apalutamide and the incidence of peri-operative outcomes and complications occurring within 30 days of RP. Biochemical relapse-free survival (bRFS) was defined as the elapsed time from study enrolment to PSA relapse (by the American Urological Association definition of PSA ≥0.2 ng/mL on two consecutive readings) and/or death due to any cause [21]. Time-to-event analysis was performed using the Kaplan-Meier method.

Tests of association were performed to explore for gene expression profiles that may be linked to biochemical and pathological response. The Wilcoxon rank-sum test was used to compare post-apalutamide outcomes between responders and non-responders. The signed-rank test was used to compare the effects of apalutamide in pre- and post-treatment tumours for the same patient. Gene-set enrichment analysis was performed on differentially expressed genes identified using DESeq2 [22]. Statistical significance was set at *P* ≤ 0.05. Multiple testing adjustment was not performed. Where appropriate, F-test was performed to compare population variances.

## Results

### Patient cohort

**Fig. 1:**
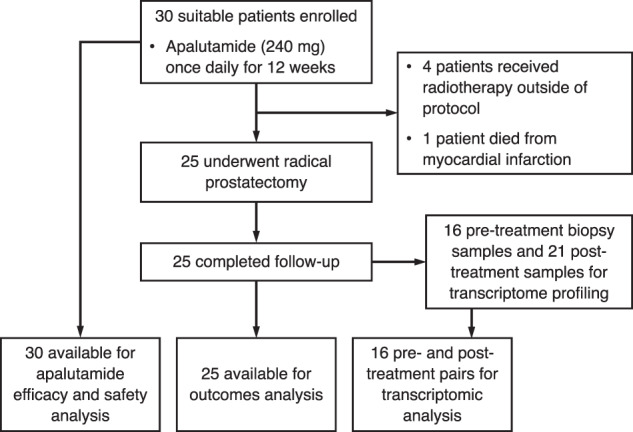
CONSORT diagram of the NEAR trial. Treatment summary and data collection of study participants.

Between June 2017 to March 2019, 30 patients were recruited. All patients completed 12 weeks of neoadjuvant apalutamide. Four patients subsequently elected for off-protocol radiotherapy, and we recorded one death prior to RP caused by acute myocardial infarction. Efficacy analysis was thus performed in 25 patients (Fig. 1).

The median age at recruitment was 68.6 (IQR: 64.8–70.9) years. The majority of patients were D’Amico high-risk (*N* = 20 [66.7%]), and 5 (16.7%) patients had ISUP Grade ≥ 4 tumours (Table 1). The median serum PSA level for the cohort was 12.8 (IQR: 9.4–22.9) ng/mL.

**Table 1:** Clinical characteristics of all 30 recruited patients and pathological and primary outcomes for 25 patients who completed the study.

Clinical Characteristics	Number of patients (%) *N* = 30 recruited
Median age at dx (IQR)	68.6 (64.8–70.9)
Race	
Chinese	27 (90.0%)
Malay	2 (6.7%)
Indian	1 (3.3%)
Prostate needle biopsy ISUP Grade Group	
1	1 (3.3%)
2	13 (43.3%)
3	11 (9.0%)
4	3 (10.0%)
5	2 (6.7%)
Median PSA at diagnosis (ng/mL) (IQR)	12.8 (9.4–22.9)
Clinical stage	
cT2	20 (66.7%)
cT3a	7 (23.3%)
cT3b	3 (10.0%)
D’Amico risk category	
Intermediate	10 (33.3%)
High	20 (66.7%)
**Treatment response**	**Number of patients (%)** *N* = 25 completed study
Pathological stage	
pT2	13 (52.0%)
pT3a	10 (40.0%)
pT3b	2 (8.0%)
Node status	
N0	21 (84.0%)
N1	4 (16.0%)
Median nodal yield (IQR)	27 (16–32)
Margin status	
Negative	21 (84.0%)
Positive	4 (16.0%)
Median change in residual cancer burden ΔCB (IQR)	−41.7% (−60.0% to −33.3%)
Pathological response grade group at surgery	
A	11 (44.0%)
B	7 (28.0%)
C	7 (28.0%)
Median ΔCB stratified by response grade group (IQR)	
A	−45.5% (−63.9% to −38.4%)
B	−41.7% (−47.5% to −14.3%)
C	−40.0% (−67.5% to −34.2%)
Biochemical response	
Achieved PSA < 0.03 ng/mL	21 (84.0%)
Above PSA ≥ 0.03 ng/mL	4 (16.0%)

### Efficacy of apalutamide

**Fig. 2:**
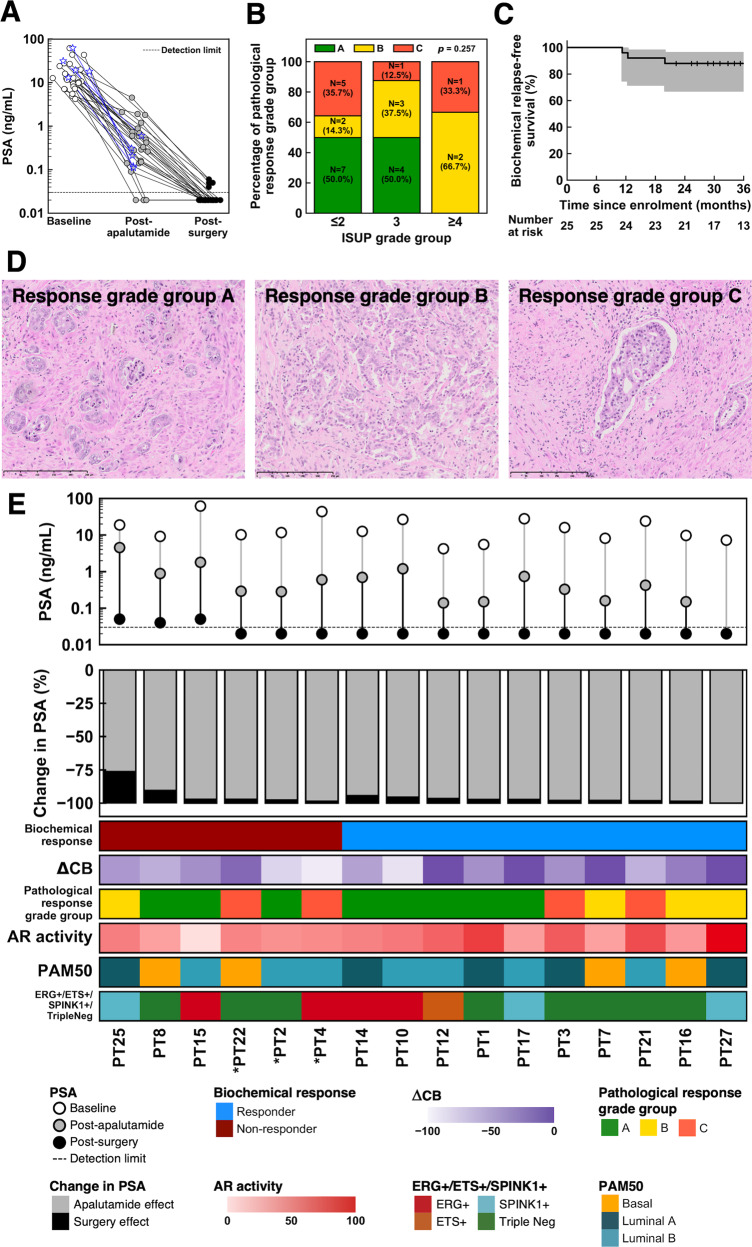
Summary of pathological and biochemical responses as well as molecular profiling of pre-treatment biopsies. **A** Spider plots of longitudinal PSA changes for 30 patients who completed neoadjuvant apalutamide. The five patients who did not undergo surgery are indicated in blue. **B** Associations of pathological response grade groups with ISUP grade groups. P-value was from Kruskal-Wallis test. **C** Kaplan-Meier curve of biochemical relapse-free survival in our cohort. Grey shaded regions indicate the 95% confidence intervals. **D** Microscopic morphology of post-apalutamide prostate cancer for pathological grade groups A, B, and C. Grade A is the most favourable response grade group, while grade C is the least favourable with the presence of cribriform glands and/or intraductal spread of tumour cells. **E** Summary of PSA changes (raw and percentages), biochemical and pathological responses (change in cancer burden [ΔCB] and response grade group). Molecular subtypes (androgen receptor activity [AR activity]; ETS + /ETS + /SPINK1 + /Triple negative subtypes and PAM50 subtypes) derived from pre-treatment transcriptome profiles are indicated. Patients who are labelled as non-responders due to early biochemical recurrence are indicated with an asterisk (*).

A total of 27 (90.0%) of 30 patients demonstrated ≥ 90% PSA response at the end of the 12-week course of apalutamide (Fig. 2A, E). Of the 25 patients who underwent RP, 21 (84.0%) achieved PSA of <0.03 ng/mL at week 24.

Median time from the last dose of apalutamide to RP was 33 (IQR: 27–40) days. Pathological staging by the AJCC 9^th^ edition indicated 13 (52.0%) patients with pT2, 10 (40.0%) with pT3a, and 2 (8.0%) with pT3b tumours (Table 1). Nodal metastasis was detected in 4 (16.0%) patients. No patient achieved a pCR; median reduction of tumour volume was 41.7% (IQR: 33.3–60.0%). Grade A response was recorded in 11 (44.0%) patients, while 14 (56.0%) patients achieved grades B and C responses (*N* = 7 for both), independent of pre-treatment ISUP grade group and serum PSA levels (Fig. 2B, eFigure 2). Pathological response grades were not associated with ΔCB (median ΔCB: −45.5% [Grade A] vs. −41.7% [Grade B] vs. −40.0% [Grade C], *P* = 0.661 by Kruskal-Wallis test; eTable 1).

At the time of analysis, median duration of follow-up was 36.7 (IQR: 31.1–40.0) months. The four patients who did not achieve a PSA of < 0.03 ng/ml at week 24 had subsequently developed a rising PSA, which however did not meet the AUA definition of PSA relapse. We further recorded three (14.3%) biochemical relapses out of the remaining 21 patients. Excluding the four patients, 2-year bRFS of the cohort was 85.7% (95% CI: 70.7%–100.0%, Fig. 2C).

### Treatment-related AEs and outcomes

Treatment-related AEs were reported in 28 (93.3%) of the 30 patients; all were grade 2 and below (eTable 2). Common AEs included dry skin (*N* = 16 [53.3%]), fatigue (*N* = 10 [33.3%]) and skin rash (*N* = 9 [30.0%]). Six (20.0%) patients required a temporary 50% dose reduction between 2 and 4 weeks due to skin rash. No patient required drug stoppage. Minor ( ≤ G2) surgical complications were observed in 5 (20.0%) of the 25 patients who underwent RP (4 developed lower urinary tract infection and 1 had obturator neuropraxia, eTable 2).

### Molecular correlates of response

**Fig. 3:**
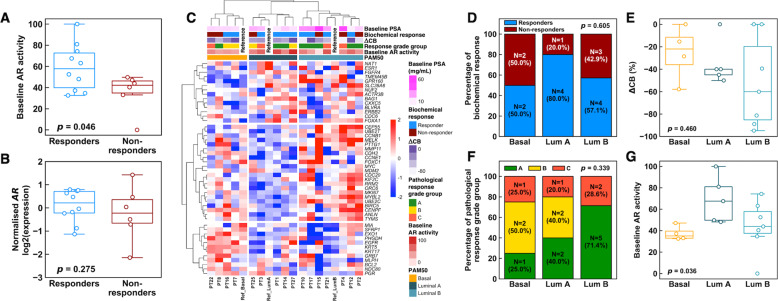
Transcriptomic profiling of pre-treatment samples (*N* = 16). **A** Baseline AR activity is significantly lower in non-responders than responders. **B** Normalised mRNA expression of *AR* for biochemical responders and non-responders. **C** Expression profile of PAM50 genes, including reference expression profiles for basal, luminal A, and luminal B molecular subtypes. Samples were hierarchically clustered (Ward’s method) by using Spearman correlation as the similarity metric, then assigned to their subtypes based on the reference profiles. Normalised gene expression values are shown from blue to red as indicated by the colour bar. **D** Association between PAM50 subtype and biochemical response. There was no obvious trend in biochemical response with PAM50 subtypes, though basal tumours appeared to have a higher rate of non-responders. **E** Basal subtype samples tend to have smaller magnitude of change in cancer burden (ΔCB) than luminal A/B subtypes. **F** Luminal B subtypes had the best pathological response outcomes as shown by the highest proportion of response grade group A. Basal tumours fared the worst. **G** Basal tumours generally had lower AR activity. P-values for **A** and **B** were obtained from Wilcoxon rank-sum tests, those for panels **D** and **F** were obtained from chi-squared tests, while those for **E** and **G** were from Kruskal-Wallis tests.

Next, we attempted to correlate molecular profiles to the biochemical and pathological response phenotypes. A total of 18 (72.0%) and seven (28.0%) patients were classified as biochemical responders and non-responders, respectively. For this exploratory analysis, patients who attained a PSA of <0.03 ng/mL without subsequent biochemical relapse during follow up were classified as responders; non-responders consisted of patients who (i) had a detectable PSA post-RP (*N* = 4) or (ii) had a biochemical relapse on follow-up (*N* = 3). Among them, 16 pre-treatment biopsies and 21 post-treatment RP samples were available. Figure 2E summarises the molecular subtypes for AR activity, PAM50 basal/luminal subtypes, and ERG-, ETS-, and SPINK1-status of the pre-treatment biopsy samples, matched to the biochemical and pathological responses [18–20]. We observed that biochemical non-responders harboured reduced AR activity in the pre-treatment biopsies, compared with responders *(P* = 0.046; Fig. 3A), albeit pre-treatment *AR* mRNA abundance was not statistically different between them (*P* = 0.275; Fig. 3B, eFigure 3). Gene-set enrichment analysis using hallmark gene-sets [23] also revealed that non-responders had reduced androgen response (eFigure 4). Innate immune-related pathways like allograft rejection, inflammatory response, and complement cascade were also significantly upregulated in non-responders than responders (adjusted p-values < 0.05).

Patients were classified into basal, luminal A, and luminal B subtypes based on the PAM50 classifier [19, 24] (Fig. 3C). While not statistically significant, we observed a higher proportion of biochemical non-responders and pathological grades B and C among the basal than the luminal A and B subtypes (Fig. 3D–F). AR activity was also lower in the basal than luminal tumours (Fig. 3G). Finally, we did not observe an association for clonal (ERG + /ETS + /SPINK1 + /triple negative) status with either biochemical or pathological response (eFigure 5).

### Post-apalutamide effects on PCa

**Fig. 4:**
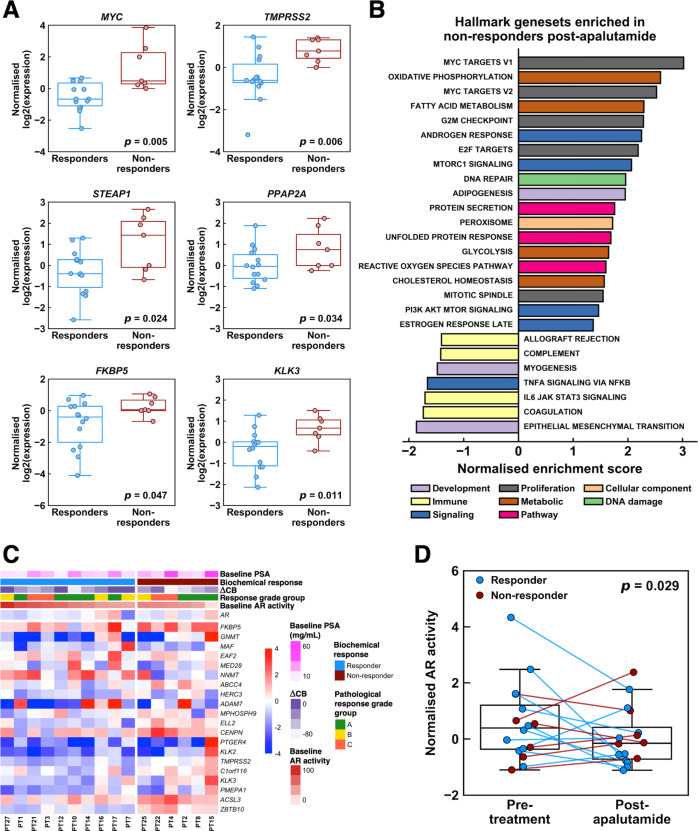
Transcriptomic profiling of post-apalutamide samples and effects of apalutamide treatment on androgen receptor activity. **A** Gene expression differences between biochemical responders and non-responders for several AR signalling pathway genes. P-values were obtained from Wilcoxon rank-sum tests. **B** Gene-set enrichment analysis between biochemical non-responders relative to responders showed an increase in MYC target pathways and androgen response, while immune pathways were downregulated in non-responders after treatment by apalutamide. **C** Log2 fold change between post-apalutamide and pre-treatment samples for genes involved in AR activity. Samples are split by biochemical response and ordered by baseline AR activity. Log2 fold change are shown from blue to red as indicated by the colour bar. **D** There is an overall decrease in AR activity after apalutamide, and non-responders had significantly less pronounced decrease in AR activity. P-value was from Wilcoxon rank-sum test comparing the differences in normalised AR activity after treatment between non-responders and responders.

We interrogated the transcriptome of the post-apalutamide RP specimens to further understand the effects of apalutamide on PCa. *TMPRSS2* expression was elevated in the RP specimens for non-responders (Fig. 4A). Other AR signalling genes were also upregulated in non-responders [25] (Fig. 4A, eFigure 6). Notably, we observed higher *MYC* expression in non-responders than responders post-apalutamide (Fig. 4A). From gene-set enrichment analyses (Fig. 4B), the androgen response and proliferation (MYC, E2F, and mitotic spindle) pathways were significantly upregulated in non-responders compared to responders, which contrasts the trends observed in the pre-treatment biopsies (eFigure 4).

Finally, we performed pairwise comparisons of transcriptomic profiles between the 16 paired samples. We observed that genes involved in AR activity were generally downregulated after apalutamide. Several genes such as *TMPRSS2*, *CENPN*, and *ZBTB10* showed differential responses to apalutamide between responders and non-responders (Fig. 4C, eFigure 7). Consistent with our earlier findings, AR activity was reduced post-apalutamide among responders, but change was minimal or increased for non-responders (Fig. 4D, eFigure 8).

## Discussion

Here, we report the results of our single-arm, phase II trial of 12 weeks of apalutamide prior to RP in 25 men with D’Amico intermediate- and high-risk localised PCa. The primary endpoint of the study was pCR, which was not observed in this trial. Nonetheless, we observed a substantial reduction in tumour volume (median of 41.7%) and 44.0% of patients with a grade A tumour response. This is corroborated by 90% of patients showing an acute PSA response of ≥ 90% from their baseline following apalutamide monotherapy. The strong clinical responses to apalutamide could have inadvertently accounted for the low surgical margin and node-positive rates (16.0% for both) in a cohort that is mostly comprised of D’Amico high-risk PCa patients. Finally, exploratory correlative analyses suggest that AR activity is linked to apalutamide response. Collectively, our trial results provide evidence for apalutamide activity in *de novo* hormone-sensitive localised PCa.

These results are consistent with other studies investigating the activity of neoadjuvant enzalutamide and abiraterone in high-risk localised PCa [9, 10]. An interim report by Sterling and colleagues investigating the addition of abiraterone acetate to ADT and apalutamide for 6 months prior to RP supports our results [26]. In that trial, investigators observed pCR and minimal residual disease rates of 3% and 10%, respectively, with combinatorial hormonal therapies. Likewise, another trial investigating the role of 6 months of enzalutamide and ADT with or without abiraterone acetate showed that triplet combination yielded a high pCR and minimal residual disease rate of 30% [12]. These results suggest that combinatorial therapy given over a longer duration is required to induce a more profound pathological response. The ongoing phase III PROTEUS trial comparing the use of ADT with and without apalutamide before and after RP will yield further insights into the optimal neoadjuvant therapy in localised high-risk PCa [27].

Next, we interrogated for potential molecular correlates of apalutamide response in our cohort. We observed that biochemical non-responders (who comprised of patients who did not attain an undetectable PSA post-RT or had a PSA relapse) harboured PCa with a lower AR activity than responders, which was consistent with previous findings showing a negative association between AR activity and response to enzalutamide [28]. Additionally, we explored the association between PAM50 basal/luminal status and biochemical response to apalutamide. This was based on previous reports indicating that luminal B PCa was more aggressive than luminal A and basal PCa, but was more likely to respond to ADT [24]. We observed that biochemical non-responders were more likely to harbour basal and luminal B tumours, and basal tumours exhibited lower AR activity than luminal tumours. These findings are consistent with the recent analyses of the SPARTAN trial, in which investigators observed a correlation between basal status and a poorer response to apalutamide in patients with castrate-resistant PCa [29].

Several limitations deserve mention. This was a single-arm study testing the efficacy of apalutamide monotherapy, and treatment with the drug alone was unable to yield any pCR, thus failing to reject the null hypothesis. Additionally, our assumption of 25% for pCR with apalutamide alone was overly optimistic, considering the results with neoadjuvant enzalutamide [9]. The small sample size also limits generalisation of our findings. Another limitation of our study included the gap between the last apalutamide dosing to the time of RP. However, a wash-out period was required by our ethics committee due to concerns of increased peri-operative complications with neoadjuvant apalutamide. Several factors could have confounded the results of the correlative biomarker analyses, including intra-tumoral spatial molecular heterogeneity [30]. The unexpected sample attrition due to low residual tumour volume in four patients would have also led to an underrepresentation of strong responders in the post-treatment samples.

## Conclusions

Herein, we report that neoadjuvant apalutamide monotherapy followed by RP failed to yield any pCR in intermediate- and high-risk localised PCa. This approach should not be taken forward for further investigation. A longer duration of combinatorial therapy before RP is likely required for an optimal tumour response, and we eagerly await the results of the PROTEUS trial (NCT03767244).

## Supplementary information

Supplementary Appendix

Supplementary- ARN509 - Clinical Trial Protocol

## Data Availability

Requests for data can be addressed to the corresponding author (LLS).
