# Correction to: NEAR trial: A single-arm phase II trial of neoadjuvant apalutamide monotherapy and radical prostatectomy in intermediate- and high-risk prostate cancer

**DOI:** 10.1038/s41391-022-00522-9

**Published:** 2022-03-14

**Authors:** Lui Shiong Lee, Adelene Y. L. Sim, Chee Wee Ong, Xinyan Yang, Cedric C. Y. Ng, Wei Liu, Vikneswari Rajasegaran, Abner M. S. Lim, Edwin Jonathan Aslim, Nye-Thane Ngo, Li-Yan Khor, Ravindran Kanesvaran, John Carson Jr Allen, Kae Jack Tay, John Shyi Peng Yuen, Tsung Wen Chong, Sun Sien Henry Ho, Bin Tean Teh, Melvin L. K. Chua

**Affiliations:** 1 Department of Urology, Sengkang General Hospital, Singapore, Singapore; 2 Duke-NUS Medical School, Singapore, Singapore; 3 Division of Medical Sciences, National Cancer Centre Singapore, Singapore, Singapore; 4 Department of Urology, Singapore General Hospital, Singapore, Singapore; 5 Department of Anatomic Pathology, Singapore General Hospital, Singapore, Singapore; 6 Division of Medical Oncology, National Cancer Centre Singapore, Singapore, Singapore; 7 Center for Quantitative Medicine/Office of Research, Duke-NUS Medical School, Singapore, Singapore; 8 Division of Radiation Oncology, National Cancer Centre Singapore, Singapore, Singapore

**Keywords:** Prostate cancer, Cancer therapy

Correction to: *Prostate Cancer and Prostatic Diseases* 10.1038/s41391-022-00496-8, published online 28 January 2022

The article "NEAR trial: A single-arm phase II trial of neoadjuvant apalutamide monotherapy and radical prostatectomy in intermediate- and high-risk prostate cancer", written by Lui Shiong Lee, Adelene Y. L. Sim, Chee Wee Ong, Xinyan Yang, Cedric C. Y. Ng, Wei Liu, Vikneswari Rajasegaran, Abner M. S. Lim, Edwin Jonathan Aslim, Nye-Thane Ngo, Li-Yan Khor, Ravindran Kanesvaran, John Carson Jr Allen, Kae Jack Tay, John Shyi Peng Yuen, Tsung Wen Chong, Sun Sien Henry Ho, Bin Tean Teh and Melvin L. K. Chua, was originally published electronically on the publisher’s internet portal on 28. January 2022 without open access. With the author(s)’ decision to opt for Open Choice the copyright of the article changed on 9. February 2022 to © The Author(s) 2022 and the article is forthwith distributed under a Creative Commons Attribution 4.0 International License, which permits use, sharing, adaptation, distribution and reproduction in any medium or format, as long as you give appropriate credit to the original author(s) and the source, provide a link to the Creative Commons licence, and indicate if changes were made.

The images or other third party material in this article are included in the article’s Creative Commons licence, unless indicated otherwise in a credit line to the material. If material is not included in the article’s Creative Commons licence and your intended use is not permitted by statutory regulation or exceeds the permitted use, you will need to obtain permission directly from the copyright holder.

To view a copy of this licence, visit http://creativecommons.org/licenses/by/4.0/. Open Access funding enabled and organized by Projekt DEAL.

