# Supplementary material for: NEAR trial: A single-arm phase II trial of neoadjuvant apalutamide monotherapy and radical prostatectomy in intermediate- and high-risk prostate cancer
Source: Prostate Cancer Prostatic Dis. 2022 Jan 28;25(4):741–8. doi: 10.1038/s41391-022-00496-8 (PMC9705244; doi:10.1038/s41391-022-00496-8)
Supplement: Supplementary file 1 — Supplementary Appendix [file 41391_2022_496_MOESM1_ESM.docx]

**Supplementary Appendix**

Lee LS, Sim AY, Ong CW, *et al.* NEAR trial: A Single-arm Phase II trial of Neoadjuvant Apalutamide Monotherapy and Radical Prostatectomy in Intermediate- and High-risk Prostate Cancer

p2. Supplementary Figures

p2. **eFigure 1. Calculation of tumour burden pre- and post-apalutamide.**

p3. **eFigure 2. Association between pre-treatment PSA and biochemical and pathological responses.**

p4. **eFigure 3. Gene expression differences of pre-treatment tumours between responders and non-responders.**

p5. **eFigure 4. Gene-set enrichment analyses between responders and non-responders.**

p6. **eFigure 5. Association between tumour clonal status and response with androgen receptor activity.**

p7. **eFigure 6. Gene expression differences of post-apalutamide tumours between responders and non-responders.**

p8. **eFigure 7. Pairwise gene expression comparison of pre- and post-apalutamide samples.**

p9. **eFigure 8. Correlation between pre- and post-apalutamide AR activity.**

p10. Supplementary Tables

p10. **eTable 1. Tumour volume, percentages and pathological grade group responses of patients who underwent radical prostatectomy.**

p11.  **eTable 2. Treatment-related adverse events for the 30 recruited patients and surgical complications for the 25 subjects who completed the study.**

**Supplementary figures**

**
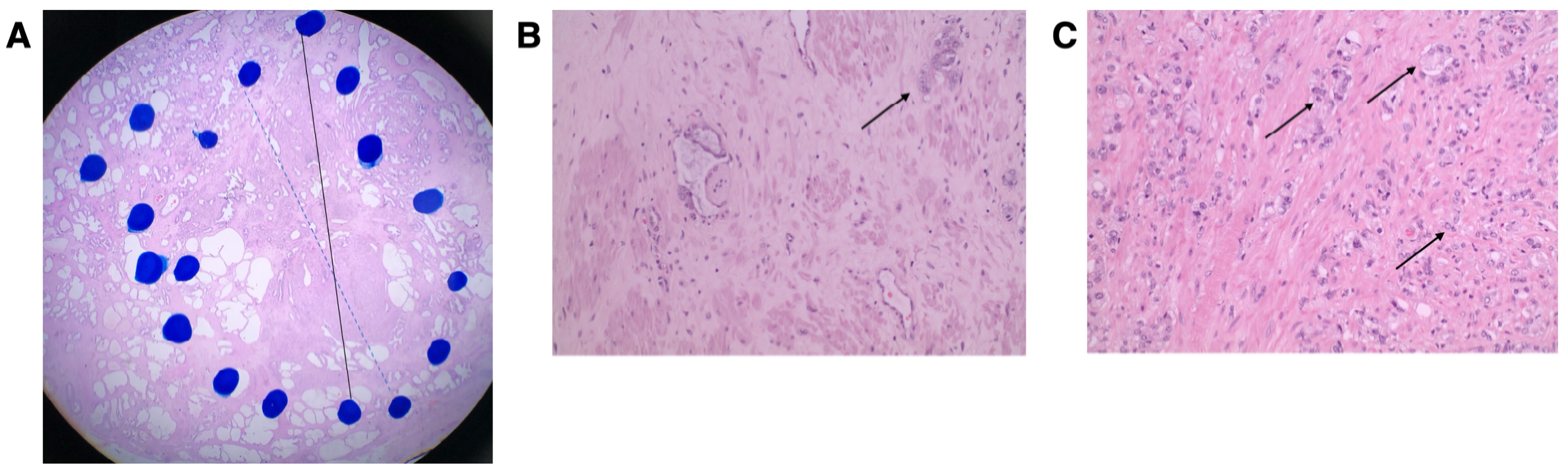
**

**eFigure 1. Calculation of tumour burden pre- and post-apalutamide.**

To determine tumour dimensions, specimens were handled in a manner previously described^1^ and tumour margins marked out under low power microscopy. The index lesion was identified and defined as the largest lesion observed on microscopy. The tumour length d1, width d2, height d3 were measured and raw tumour volume (TV) calculated using the formula^2^ π/6*d1*d2*d3, where π=3.142. The percentage of viable tumour (TP) within the index lesion was estimated under low power light microscopy, and the corrected tumour volume calculated using TP (%) x TV (mm^3^).

(An assumption was made that tumour dimensions remained constant before and after apalutamide). **(A)** Estimation of residual cancer burden dimensions via light microscopy. Example of a tumour with 5% cellularity **(B)** and 45% cellularity **(C)**. Arrow denotes residual cancer cells.

**
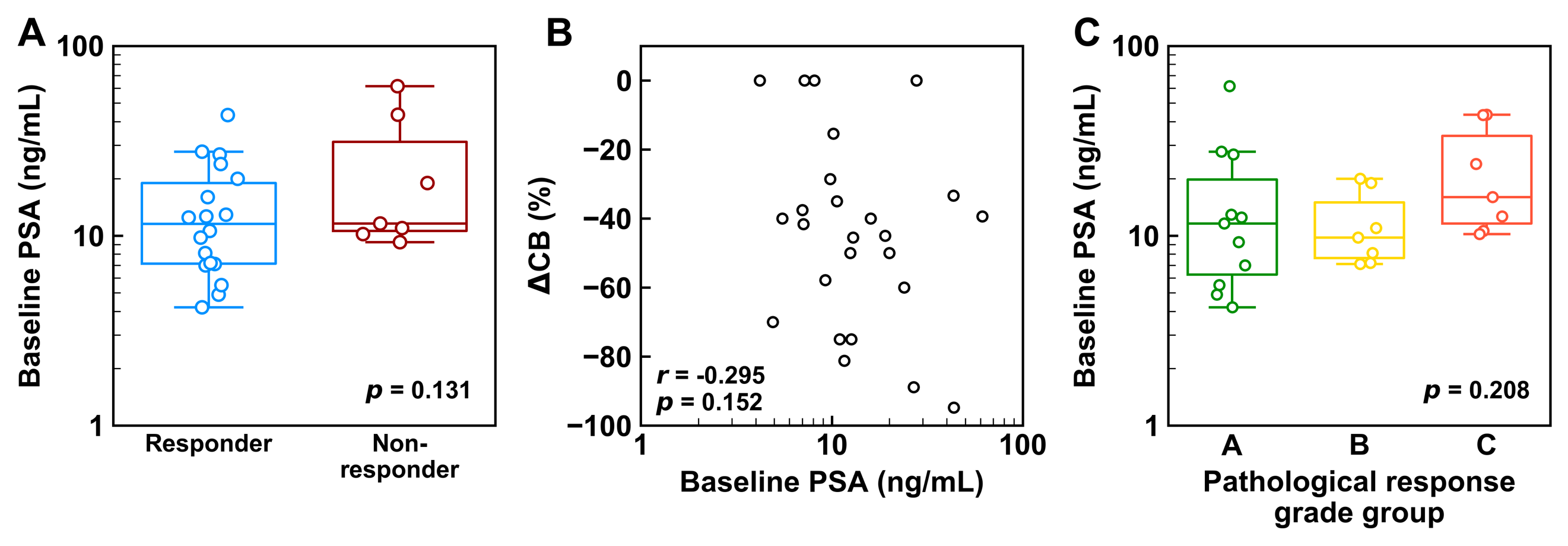
**

**eFigure 2. Association between pre-treatment PSA and biochemical and pathological responses.**

Association between pre-treatment PSA and biochemical response **(A)**, change in cancer burden (ΔCB) **(B)** and pathological response grade group **(C)**. P-values were evaluated using Wilcoxon rank-sum, Spearman correlation and Kruskal-Wallis tests, respectively.

**
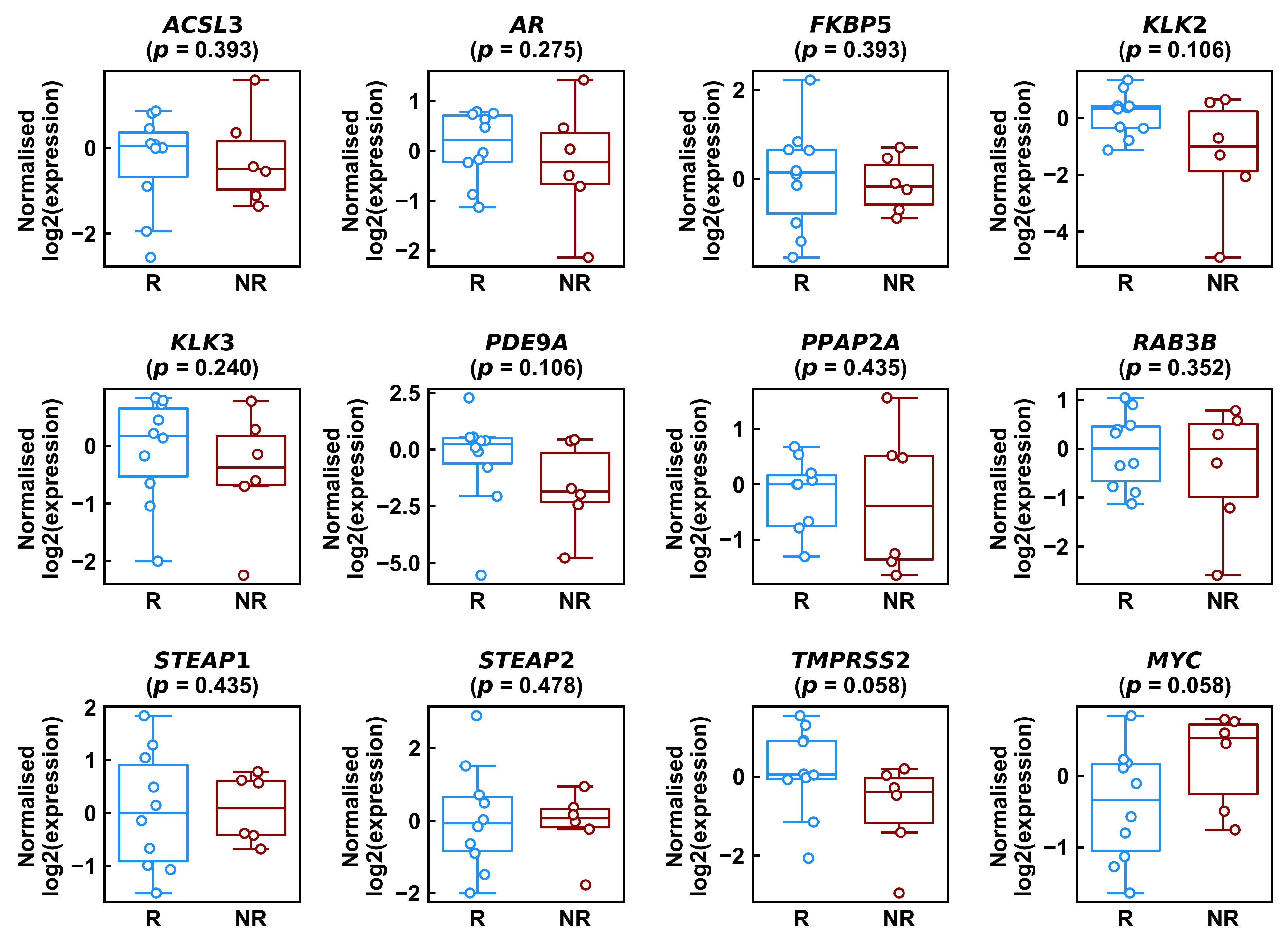
**

**eFigure 3. Gene expression differences of pre-treatment tumours between responders and non-responders.**

Normalised gene expression of key genes in pre-treatment samples for responders (R) and non-responders (NR).

**
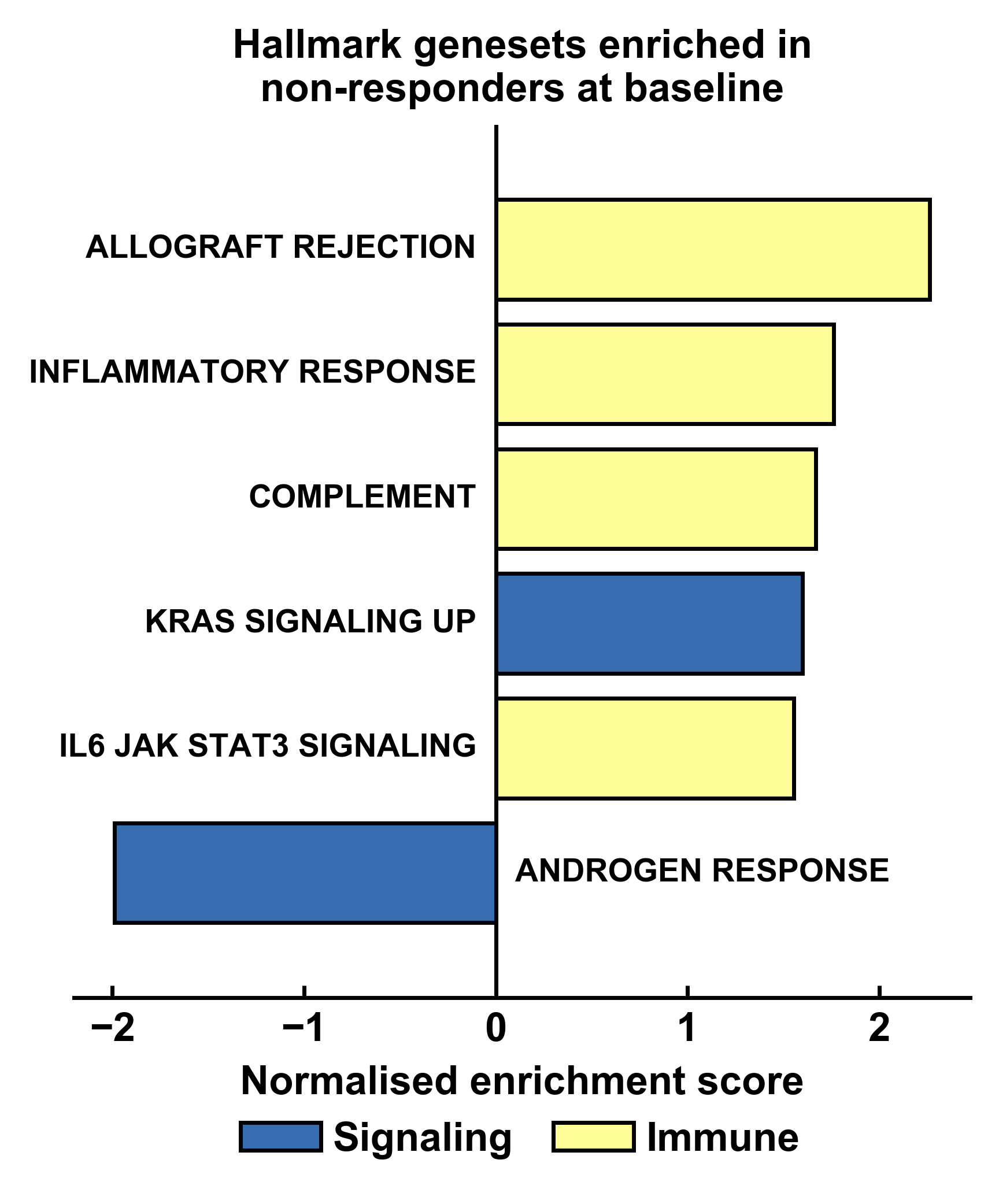
**

**eFigure 4. Gene-set enrichment analyses between responders and non-responders.**

Immune processes are upregulated in non-responders while the androgen response pathway is significantly downregulated. Only hallmark gene-set pathways^3^ with adjusted p-values <0.05 are shown.

**
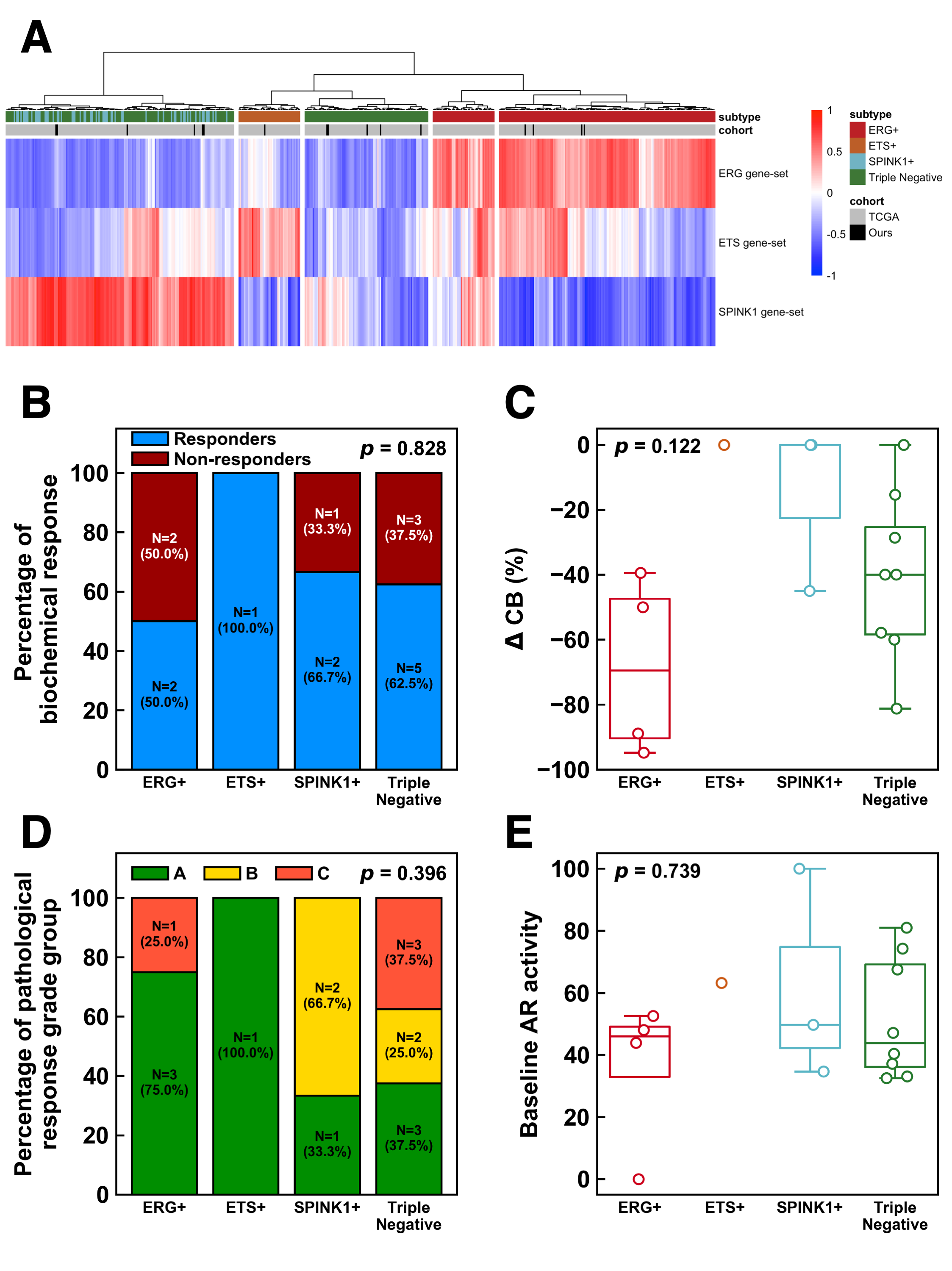
**

**eFigure 5. Association between tumour clonal status and response with androgen receptor activity.**

**(A)** ERG+, ETS+, SPINK1+ and Triple Negative subtype assignments^4^ based on co-clustering of our cohort with the TCGA prostate cancer cohort. Clustering was performed on single sample gene-set variation scores for each subtype^5^. The subgroup of tumours with enriched SPINK1 gene-set expression were further separated into SPINK1+ and Triple Negative subtypes based on *SPINK1* expression. (**B**) Association between subtypes and biochemical response, (**C**) change in cancer burden (ΔCB), (**D**) pathological response grade group, and (**E**) baseline androgen receptor (AR) activity. P-values were obtained from chi-squared and Wilcoxon rank-sum tests.

**
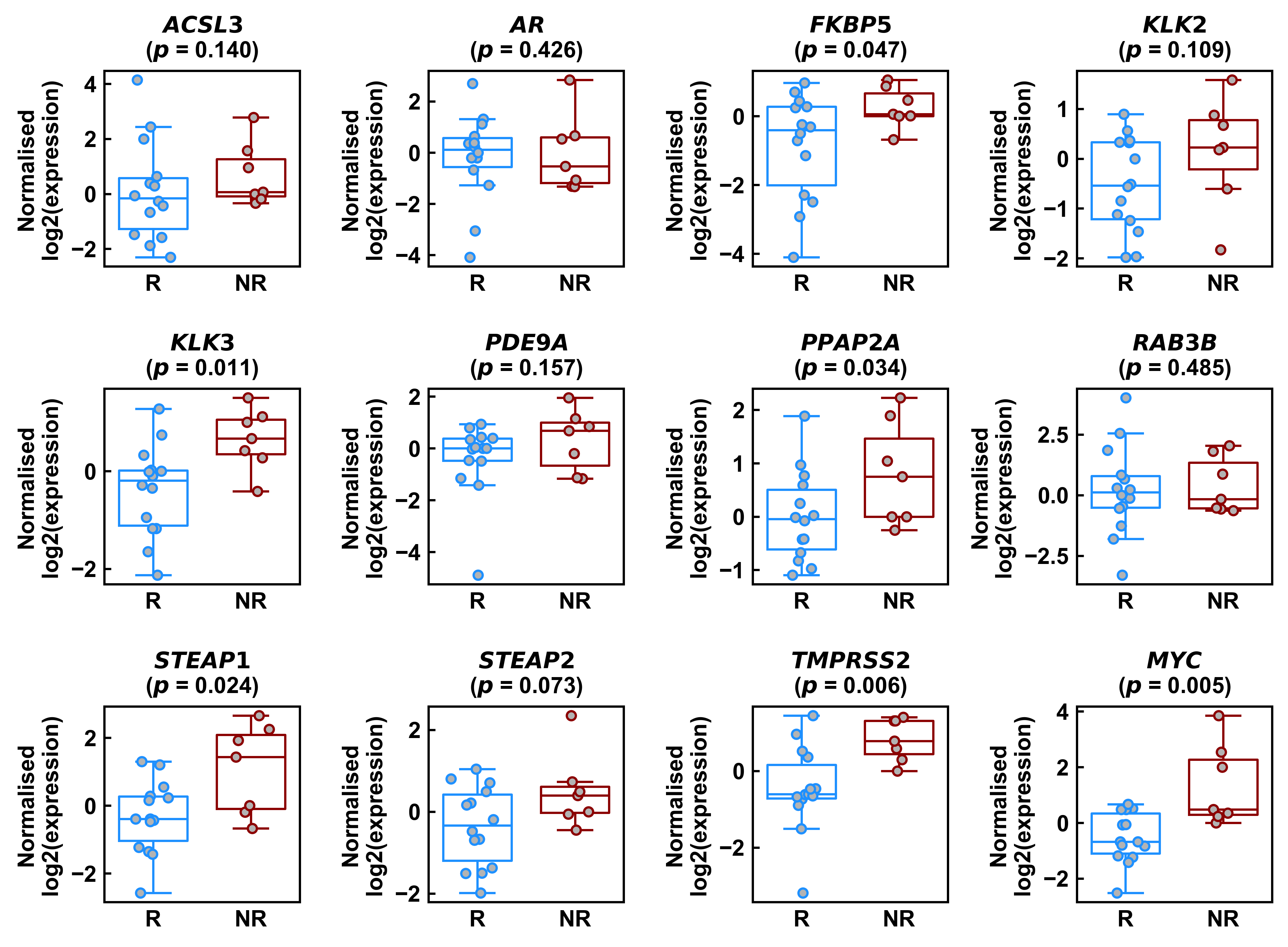
**

**eFigure 6. Gene expression differences of post-apalutamide tumours between responders and non-responders.**

Normalised gene expression of key genes in post-apalutamide samples for responders (R) and non-responders (NR). P-values obtained by Wilcoxon rank-sum tests are indicated.


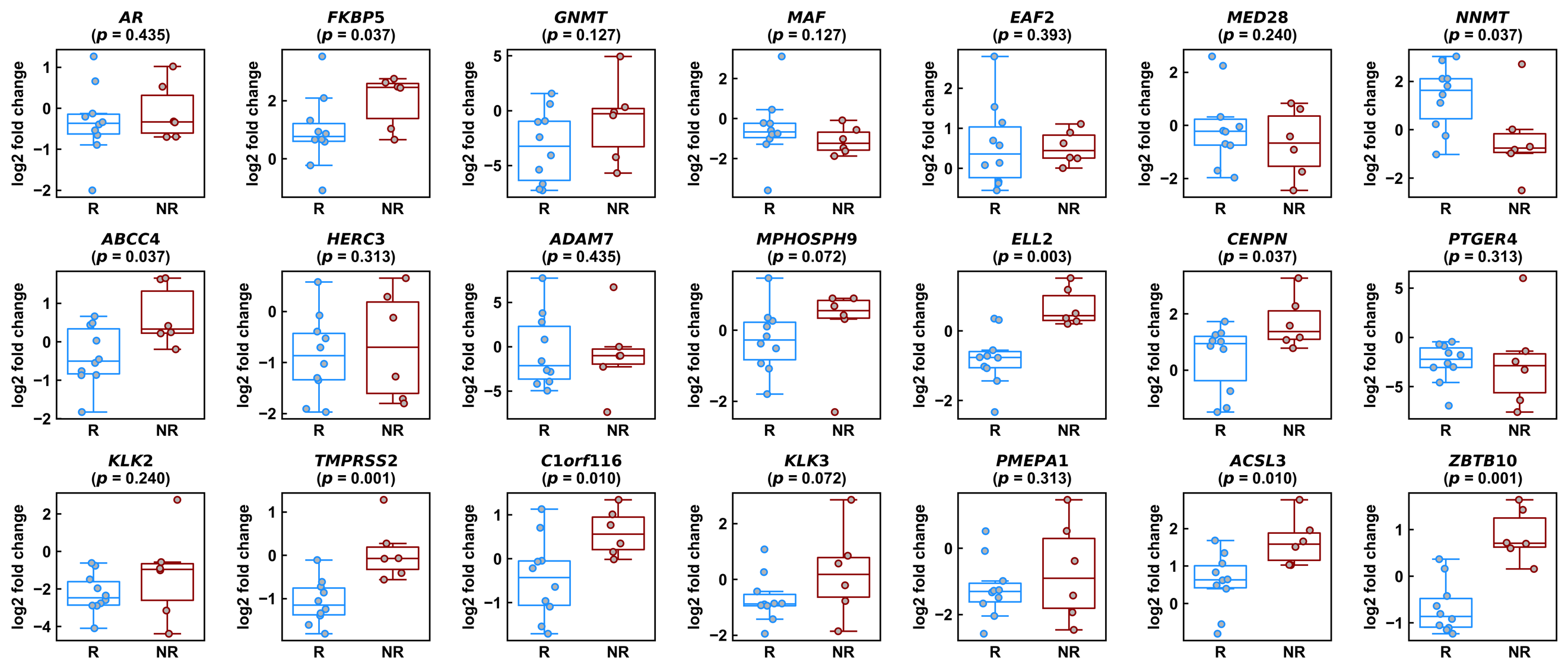


**eFigure 7. Pairwise gene expression comparison of pre- and post-apalutamide samples.**

Log2 fold change in gene expression of androgen receptor (AR) activity genes between pre- and post-apalutamide tumours for responders (R) and non-responders (NR). A log2 fold change of 0 indicates no change in expression before and after apalutamide, while a negative (positive) log2 fold change is indicative a decrease (an increase) in expression after apalutamide treatment. P-values are obtained from Wilcoxon rank-sum tests.

**
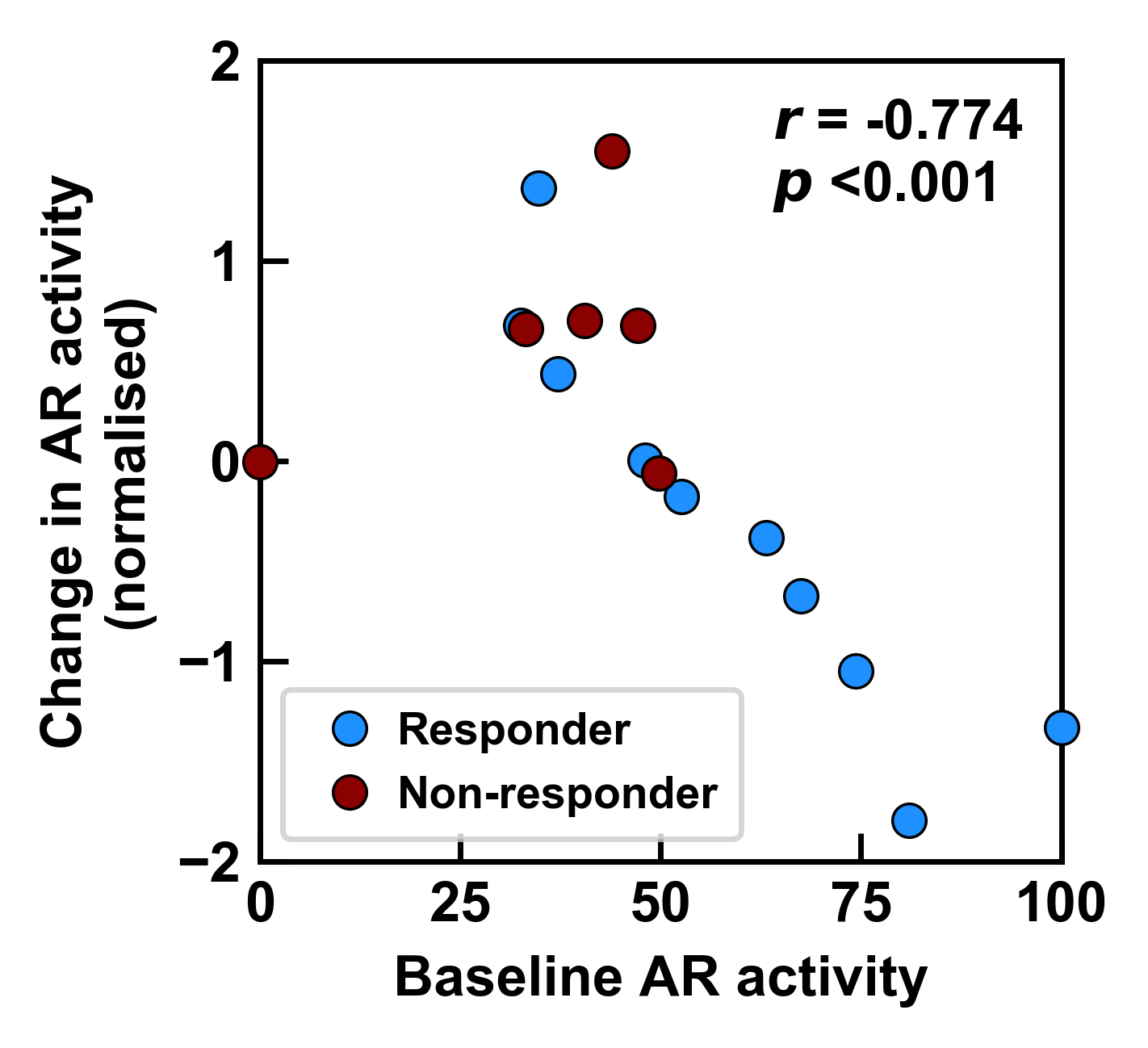
**

**eFigure 8. Correlation between pre- and post-apalutamide AR activity.**

Baseline androgen receptor (AR) activity is negatively correlated with change in AR activity after 12 weeks of apalutamide treatment. Spearman correlation and associated p-value are shown.

**Supplementary Table**

**eTable 1: Tumour volume, percentages and grade group responses of the 25 patients who underwent radical prostatectomy.**

| Subject | Tumour  dimension d1, mm | Tumour dimension d2, mm | Tumour dimension d3, mm | Pre-treatment tumour percentage, pre- TP (%) | Post treatment tumour percentage, post-TP (%) | Pre-treatment tumour volume, pre-TV(calculated) | Post-treatment tumour volume, post-TV (calculated) | Relative change in tumour volume, ΔCB (%) | Grade group response |
| --- | --- | --- | --- | --- | --- | --- | --- | --- | --- |
| ARN1 | 18 | 11 | 28 | 75 | 45 | 74 | 45 | -40 | A |
| ARN2 | 5.0 | 3.0 | 20 | 80 | 15 | 6 | 1.1 | -81 | A |
| ARN3 | 19 | 11 | 21 | 50 | 30 | 52 | 31 | -40 | C |
| ARN4 | 40 | 25 | 27 | 95 | 5 | 475 | 25 | -95 | C |
| ARN5 | 5.0 | 8.0 | 13 | 50 | 15 | 10 | 3 | -70 | A |
| ARN6 | 8.0 | 7.0 | 14 | 10 | 65 | 28 | 18 | -35 | C |
| ARN7 | 5.0 | 5.0 | 15 | 30 | 30 | 3.8 | 3.8 | 0.0 | B |
| ARN8 | 19 | 28 | 24 | 95 | 40 | 253 | 106 | -58 | A |
| ARN10 | 13 | 21 | 21 | 90 | 10 | 123 | 14 | -89 | A |
| ARN11 | 18 | 15 | 28 | 10 | 25 | 135 | 34 | -75 | B |
| ARN12 | 6.0 | 13 | 19 | 25 | 25 | 9.8 | 9.8 | 0.0 | A |
| ARN13 | 6.0 | 18 | 15 | 80 | 50 | 43 | 27 | -38 | A |
| ARN14 | 15 | 13 | 28 | 90 | 45 | 88 | 44 | -50 | A |
| ARN15 | 31 | 10 | 20 | 66 | 40 | 102 | 62 | -39 | A |
| ARN16 | 9.0 | 10 | 15 | 70 | 50 | 32 | 23 | -29 | B |
| ARN17 | 18 | 12 | 13 | 40 | 40 | 43 | 43 | 0.0 | A |
| ARN18 | 10 | 5.0 | 24 | 60 | 15 | 15 | 3.8 | -75 | C |
| ARN19 | 3.0 | 5.0 | 6 | 55 | 30 | 4.1 | 2.3 | -45 | A |
| ARN21 | 30 | 17 | 21 | 10 | 40 | 255 | 102 | -60 | C |
| ARN22 | 25 | 7.0 | 15 | 65 | 55 | 57 | 48 | -15 | C |
| ARN24 | 10 | 15 | 13 | 60 | 35 | 45 | 26 | -42 | B |
| ARN25 | 31 | 25 | 36 | 10 | 55 | 388 | 213 | -45 | B |
| ARN27 | 8.0 | 10 | 15 | 45 | 45 | 18 | 18 | 0.0 | B |
| ARN28 | 6 | 5 | 3.5 | 0.6 | 0.3 | 9.0 | 4.5 | -50 | B |
| ARN30 | 15 | 11 | 18 | 0.6 | 0.4 | 50 | 33 | -33 | C |

**eTable 2: Treatment-related adverse events for the 30 recruited patients and surgical complications for the 25 subjects who completed the study.** CTCAE: Common Terminology Criteria for Adverse Events.

| **Treatment-related adverse events (TRAEs)** | **Number of patients (%)**  N = 30 completed 12 weeks of apalutamide |
| --- | --- |
| Any TRAE | 28 (93.3%) |
| Significant TRAE (CTCAE grade 3 and above) | 0 (0.0%) |
| Most commonly reported adverse events  Dry skin  Fatigue  Rashes  Gynaecomastia and nipple area discomfort  Weight loss | 16 (53.3%)  10 (33.3%)  9 (30.0%)  7 (23.3%)  4 (13.3%) |
| TRAE leading to drug dose modification  Rashes  Dry skin  Concurrent upper respiratory tract infection | 5 (16.7%)  1 (3.3%)  1 (3.3%) |
| **Surgical complications** | **Number of patients (%)**  N = 25 completed study |
| Surgical complication by Clavien-Dindo grade  None  1-2  3 and above | 20 (80.0%)  5 (20.0%)  0 (0.0%) |

**References**

1. Alvin LW, Gee SH, Hong HH, Christopher CW, Henry HS, Weber LK, et al. Oncological outcomes following robotic-assisted radical prostatectomy in a multiracial Asian population. Journal of robotic surgery. 2015;9(3):201-9.

2. Noguchi M, Stamey TA, McNeal JE, Yemoto CE. Assessment of morphometric measurements of prostate carcinoma volume. Cancer. 2000;89(5):1056-64.

3. Liberzon A, Birger C, Thorvaldsdóttir H, Ghandi M, Mesirov Jill P, Tamayo P. The Molecular Signatures Database Hallmark Gene Set Collection. Cell Systems. 2015;1(6):417-25.

4. Tomlins SA, Alshalalfa M, Davicioni E, Erho N, Yousefi K, Zhao S, et al. Characterization of 1577 Primary Prostate Cancers Reveals Novel Biological and Clinicopathologic Insights into Molecular Subtypes. European Urology. 2015;68(4):555-67.

5. Hänzelmann S, Castelo R, Guinney J. GSVA: gene set variation analysis for microarray and RNA-Seq data. BMC Bioinformatics. 2013;14(1):7.
