# Supplementary material for: NEAR trial: A single-arm phase II trial of neoadjuvant apalutamide monotherapy and radical prostatectomy in intermediate- and high-risk prostate cancer
Source: Prostate Cancer Prostatic Dis. 2022 Jan 28;25(4):741–8. doi: 10.1038/s41391-022-00496-8 (PMC9705244; doi:10.1038/s41391-022-00496-8)
Supplement: Supplementary file 2 — Supplementary- ARN509 - Clinical Trial Protocol [file 41391_2022_496_MOESM2_ESM.doc]

**CLINICAL TRIAL PROTOCOL**

| **PROTOCOL TITLE:** | |
| --- | --- |
| [Neoadjuvant apalutamide (ARN509) and radical prostatectomy in treatment of intermediate to high risk prostate cancer](http://ishareintranet/Workspace.aspx?studyid=3dfb7248-ddf9-4028-a12c-bd0f86487efa) | |
|  | |
| **PROTOCOL NUMBER:** | |
| ARN509 - 2016 | |
|  | |
| **PROTOCOL VERSION:** | 2.0 |
| **PROTOCOL DATE:** | 10 Apr 2017 |
|  | |
| **PRINCIPAL INVESTIGATOR:** | |
| Lee Lui Shiong, Consultant, Department of Urology, SGH | |
|  | |

Table of Contents

[1 BACKGROUND AND RATIONALE 5](#__RefHeading___Toc365634317)

[1.1 General Introduction 5](#__RefHeading___Toc365634318)

[1.2 Rationale and Justification for the Study 5](#__RefHeading___Toc365634319)

[1.2.1 Rationale for the Study Purpose 5](#__RefHeading___Toc365634320)

[1.2.2 Rationale for Doses Selected 5](#__RefHeading___Toc365634321)

[1.2.3 Rationale for Study Population 5](#__RefHeading___Toc365634322)

[1.2.4 Rationale for Study Design 5](#__RefHeading___Toc365634323)

[2 HYPOTHESIS AND OBJECTIVES 5](#__RefHeading___Toc365634324)

[2.1 Hypothesis 5](#__RefHeading___Toc365634325)

[2.2 Primary Objectives 6](#__RefHeading___Toc365634326)

[2.3 Secondary Objectives 6](#__RefHeading___Toc365634327)

[2.4 Potential Risks and Benefits: 6](#__RefHeading___Toc365634328)

[2.4.1 Potential Risks 6](#__RefHeading___Toc365634329)

[2.4.2 Potential Benefits 6](#__RefHeading___Toc365634330)

[3 STUDY POPULATION 6](#__RefHeading___Toc365634331)

[3.1 List The Number and nature of Subjects to be Enrolled. 6](#__RefHeading___Toc365634332)

[3.2 Criteria for Recruitment and Recruitment Process 6](#__RefHeading___Toc365634333)

[3.3 Inclusion Criteria 6](#__RefHeading___Toc365634334)

[3.4 Exclusion Criteria 7](#__RefHeading___Toc365634335)

[3.5 Subject Replacement 7](#__RefHeading___Toc365634336)

[4 STUDY DESIGN 7](#__RefHeading___Toc365634337)

[4.1 Randomisation and Blinding 7](#__RefHeading___Toc365634338)

[4.2 Contraception and Pregnancy Testing 7](#__RefHeading___Toc365634339)

[4.3 Study Visits and Procedures 7](#__RefHeading___Toc365634340)

[4.3.1 Screening Visits and Procedures 8](#__RefHeading___Toc365634341)

[4.3.2 Study Visits and Procedures 8](#__RefHeading___Toc365634342)

[4.3.3 Final Study Visit: 8](#__RefHeading___Toc365634343)

[4.3.4 Post Study Follow up and Procedures 8](#__RefHeading___Toc365634344)

[4.4 Discontinuation/Withdrawal 8](#__RefHeading___Toc365634345)

[4.4.1 Discontinuation Criteria 8](#__RefHeading___Toc365634346)

[4.4.2 Discontinuation Visit and Procedures 8](#__RefHeading___Toc365634347)

[5 TRIAL MATERIALS 8](#__RefHeading___Toc365634348)

[5.1 Trial Product (s) 9](#__RefHeading___Toc365634349)

[5.2 Storage and Drug Accountability 9](#__RefHeading___Toc365634350)

[6 TREATMENT 9](#__RefHeading___Toc365634351)

[6.1 Rationale for Selection of Dose 9](#__RefHeading___Toc365634352)

[6.2 Study Drug Formulations 9](#__RefHeading___Toc365634353)

[6.3 Study Drug Administration 9](#__RefHeading___Toc365634354)

[6.4 Specific Restrictions / Requirements 9](#__RefHeading___Toc365634355)

[6.5 Blinding 9](#__RefHeading___Toc365634356)

[6.6 Concomitant therapy 9](#__RefHeading___Toc365634357)

[7 SAFETY MEASUREMENTS 10](#__RefHeading___Toc365634358)

[7.1 Definitions 10](#__RefHeading___Toc365634359)

[7.2 Collecting, Recording and Reporting of Adverse Events and Serious Adverse Events to CIRB 10](#__RefHeading___Toc365634360)

[7.3 Collecting, Recording and Reporting of Serious Adverse Events (SAEs) to the Health Science Authority (HSA) 11](#__RefHeading___Toc365634361)

[7.4 Safety Monitoring Plan 11](#__RefHeading___Toc365634362)

[7.5 Complaint Handling 11](#__RefHeading___Toc365634363)

[8 DATA ANALYSIS 11](#__RefHeading___Toc365634364)

[8.1 Data Quality Assurance 11](#__RefHeading___Toc365634365)

[8.2 Data Entry and Storage 11](#__RefHeading___Toc365634366)

[9 SAMPLE SIZE AND STATISTICAL METHODS 11](#__RefHeading___Toc365634367)

[9.1 Determination of Sample Size 11](#__RefHeading___Toc365634368)

[9.2 Statistical and Analytical Plans 11](#__RefHeading___Toc365634369)

[10 DIRECT ACCESS TO SOURCE DATA/DOCUMENTS 12](#__RefHeading___Toc365634370)

[11 QUALITY CONTROL AND QUALITY ASSURANCE 12](#__RefHeading___Toc365634371)

[12 ETHICAL CONSIDERATIONS 12](#__RefHeading___Toc365634372)

[12.1 Informed Consent 12](#__RefHeading___Toc365634373)

[12.2 Confidentiality of Data and Patient Records 13](#__RefHeading___Toc365634374)

[13 PUBLICATIONS 13](#__RefHeading___Toc365634375)

[14 RETENTION OF TRIAL DOCUMENTS 13](#__RefHeading___Toc365634376)

[15 FUNDING and INSURANCE 13](#__RefHeading___Toc365634377)

**PROTOCOL SIGNATURE PAGE**

Protocol Title: **Neoadjuvant apalutamide (ARN509) and radical prostatectomy in the treatment of intermediate to high risk prostate cancer**

Protocol Number: ARN 509 -2016

Protocol Version/ Date: 2.0 / 10 April 2017

Sponsor Name: Singapore General Hospital

Declaration of Investigator

I confirm that I have read the above-mentioned protocol and its attachments. I agree to conduct the described trial in compliance with all stipulations of the protocol, regulations and Singapore Guideline for Good Clinical Practice (SGGCP).

| Principal Investigator Name:  ___Lee Lui Shiong________________    Principal Investigator Signature:  _______________________________________    Date:  ____10 April 2017______________________________________ |
| --- |

| **BACKGROUND AND RATIONALE** |
| --- |
| General Introduction There is no established best treatment modality for intermediate-high and high risk (D Amico) prostate cancer patients, especially with regards to the role of systemic therapy. Although there is evidence to show that conventional androgen deprivation therapy augments radiation and surgical outcomes in node positive patients, disease related morbidity and mortality can occur. Our experience has shown that following definitive radical surgery for organ confined prostate cancer, patients in these cancer risk group categories may have serum prostate specific antigen (PSA) recurrence of up to 50%, which would entail additional therapy .  Previous studies on neoadjuvant chemotherapy report oncological benefits but are associated with significant side effects , which has hindered its establishment as routine clinical practice.  Neoadjuvant androgen deprivation therapy (ADT) with luteininsing hormone agonists (goserelin) or partial AR receptor antagonists ( bicalutamide, flutamide) has also been shown to decrease margin positive rates after surgery but there is no additional benefit towards better long term disease control .  The lack of clinical efficacy of systemic testosterone suppression may be due to the inadequacy of testosterone suppression with conventional androgen deprivation therapy (ADT), and this is also supported by the findings of adverse cancer outcomes with inadequate castration in metastatic disease .  Attempts to totally suppress circulating testosterone with combined LHRH agonists and abiraterone have led to more effective pathological response at prostatectomy in phase II studies.  However, tumour cells may also synthesize intratumoural androgens that can trigger androgen receptor (AR) transcription via conventional ligand binding. This permits tumour cellular proliferation in an androgen deprived environment.  Therefore, approaches to block androgen receptor mediated gene transcription that drive prostate cancer oncognesis and progression have been attempted as a method for neoadjuvant treatment. Older AR antagonists such as bicalutamide and flutamide, have limitations of in-vivo partial agnostic activity at the AR ligand binding domain (LBD) in vitro, and may attenuate clinical efficacy in neoadjuvant studies.  Newer generation AR antagonists such as ARN 509(apalutmaide) possess a higher affinity for the LBD and exert near total antagonists activity. In addition to antagonism at the LBD of the AR, ARN509 also prevented AR mediated transcription of target genes in vitro .  This is further supported in phase II clinical studies showing additional efficacy of ARN509 in patients previously treated in conventional agents (such as bicalutmamide and flutamide). After 12 weeks of treatment with ARN509, 89% of such previously treated patients had a PSA decline of >50% from baseline.  ARN509 is currently in phase III neoadjuvant trials combined with radiation therapy for organ confined prostate cancer, and as primary systemic therapy in metastatic castrate sensitive prostate cancer.  **Gaps in clinical practice**  Optimal local and systemic cancer control is most efficacious when the total systemic burden of disease is low. Yet, there is no established treatment regime combining neoadjuvant therapy with radical surgery in organ confined prostate cancer. There is also a paucity of agents that demonstrate minimal systemic side effects that would be easily acceptable by most patients.  The standard therapy of radical prostatectomy is efficacious but not optimal for intermediate to high risk prostate cancer where there is a risk of PSA recurrence of up to 50%. In a select group of patients with high risk prostate cancer, about 25% of those with upfront surgical treatment do not achieve nadir PSA immediately after , and require adjuvant therapy for optimal disease control.  PSA recurrence after radical prostatectomy is associated with a significant risk of prostate cancer metastasis and mortality in long term studies.  Therefore, there is an imperative need to augment the clinical efficacy of radical surgery for long term cancer control. A large focus of the urological community is finding the optimal combination of neoadjuvant therapy combined with surgery, which is an approach adopted in other cancer types treated surgically (eg. Colorectal cancer, bladder cancer, breast cancer).  Therefore, there is an imperative need for establishing an acceptable and efficacious neoadjuvant therapeutic treatment modality combined with radical surgery in intermediate to high risk organ confined prostate cancer. |
| Rationale and Justification for the Study The choice of dosage and duration of neoadjuvant therapy is based on preceding Phase I and II studies on ARN509 in castrate resistant disease.  Phase I studies on ARN509 demonstrate androgen receptor blockade on imaging at 4 weeks of continuous oral administration. Phase II studies prescribe a daily oral administration of 240mg of ARN509. The steady state levels in serum is achieved at 43 weeks of continuous oral treatment.  Although treatment duration of ARN 509 is 3 months in phase I and II studies, there is no other available evidence to suggest optimal duration in the neoadjuvant setting where the burden of systemic disease is limited.  In addition, when ARN 509 is used in the CRPC setting where patients have been heavily pre-treated prior to entry into the trial, it is difficult to extrapolate the optimal duration required in treatment naïve patients. However, it is reasonable to assume that the absence of prior androgen deprivation therapy (ADT) or a short duration of prior ADT will allow full ARN509 activity on existing tumours which would be highly sensitive to androgen-receptor specific inhibition.  A duration of 12 weeks neoadjuvant therapy is chosen to allow sufficient time for androgen receptor blockade, and an achievement of a steady serum drug level. |
|  |
| Rationale for the Study Purpose |
| Optimal local and systemic cancer control is most efficacious when the total systemic burden of disease is low. Yet, there is no established treatment regime combining neoadjuvant therapy with radical surgery in organ confined prostate cancer. There is also a paucity of agents that demonstrate minimal systemic side effects that would be easily acceptable by most patients.  Therefore, there is an imperative need for establishing an acceptable and efficacious neoadjuvant therapeutic treatment modality combined with radical surgery in intermediate to high risk organ confined prostate cancer. |
| Rationale for Doses Selected |
| This is explained in section 6.1, and is based on preceding human studies. |
| Rationale for Study Population |
| Patients with low risk prostate cancer are managed largely by active surveillance initially. Therefore, they are excluded from this study.  Intermediate and high risk patients continue to benefit from radical therapy. |
| Rationale for Study Design |
| This is a phase II single arm study to look at efficacy feasibility and safety of neoadjuvant ARN 509 with radical prostatectomy. The outcomes of this study will form the background for subsequent phase III randomised trials. |
|  |
| **HYPOTHESIS AND OBJECTIVES** |
|  |
| Hypothesis |
| The hypothesis is that neoadjuvant ARN 509 combined with radical prostatectomy is a safe and feasible treatment option for organ-confined intermediate to high risk prostate cancer. |
| Primary Objectives |
| 1. Objective – to assess the proportion of patients with pathological downstaging at radical prostatectomy   (ii) Objective – to assess the  oncological efficacy of combination neoadjuvant ARN 509 and radical prostatectomy in primary prostate tumours |
| Secondary Objectives  1. Objective – to the rate of CTCAE grade 3 or high adverse events 2. Objective – to assess the proportion of patients with peri-operative Clavien-Dindo complications grade 3 and above. 3. Objective (exploratory) – to assess the effect of neoadjuvant ARN 509 on apoptosis and androgen signaling of prostate cancer |
|  |
| Potential Risks and Benefits: |
| Potential Risks |
| There have been adverse drug reactions reported in ARN 509 human studies including fatigue, decreased appetite, nausea , constipation, dysgeusia. It is not known if these are directly caused by the study drug.  Seizures have been observed in animals study at very high serum levels of the drug, but not observed in human studies utilising 240mg daily doses. |
| Potential Benefits |
| The potential direct benefits to participants include:  (i) better long term cancer control when there is synergism between neoadjuvant androgen receptor inhibition and radical surgery  (ii)  better understanding of the clinical efficacy of ARN509 in organ confined prostate cancer will potentially allow wider clinical indications for this new drug.  (iii) with better long term cancer control, there is a lower risk of recurrence and the need for future treatment including prolonged androgen deprivation therapy or radiation therapy  (iv) downstaging of disease in the prostate allows for higher probability of nerve sparing surgery which will lead to better functional outcomes of continence and potency  (v) the background information from this study may allow the study team to progress onto a multi-centre, double-blind, placebo controlled trial which will enhance the care of prostate cancer for patients  The indirect benefits to the participants include:  (i) better understanding of the biological response of human prostate cancer to ARN 509 which will potentially give insight to new therapeutic options |
|  |
| **STUDY POPULATION** |
| List The Number and Nature of Subjects to be Enrolled. |
| This will be a single centre study recruiting n=30 patients. Only male patients between 21-75 years of age will be recruited as this age range represents the surgical cohort predominant in our local practice. |
| Criteria for Recruitment and Recruitment Process |
| Suitable patients will be screened by the study team investigators and recruited in the outpatient clinics in the Singapore General Hospital. |
| Inclusion Criteria |
| The inclusion criteria include   1. those aged between 21 to 75 years of age with histologically diagnosed primary adenocarcinoma of the prostate gland within 6 months of recruitment 2. non-metastatic D’Amico intermediate to high risk patients undergoing radical prostatectomy as primary definitive therapy 3. no known hypersensitivity to the study drug 4. able to swallow study drug as whole tablets |
| Exclusion Criteria |
| The exclusion criteria will include   1. presence of small cell, neuroendocrine or ductal differentiation at needle biopsy of the prostate gland 2. individuals with prior pelvic irradiation therapy for any form of pelvic malignancy 3. patients with psychiatric conditions requiring anti-psychotic therapy, or preventing the provision of informed consent 4. renal impairment with serum creatinine more than twice the upper limit of normal 5. Other prior malignancy less than or equal to 5 years prior to recruitment 6. ECOG performance status 2 or poorer |
| Subject Replacement |
| There will be an attempt to replace subjects who drop out of the study. |
| **STUDY DESIGN** |
| This is a single arm phase II study in a single centre in Singapore.  Upon recruitment, all patients will undergo oral administration of ARN 509 (apalutamide) 240mg once daily for 12 weeks. This is followed by standard of care radical prostatectomy within 4 weeks of the last dose of ARN 509.  The details of visits and research related activity schedule is listed in Table 1. |
| There will be no video recording of these procedures. |
| Randomisation and Blinding |
| There is no randomisation or blinding in this study. |
| Contraception and Pregnancy Testing |
| If the study subject is engaged with sexual activity with a woman of childbearing potential, he should use contraception in the time period during and up to 3 months after the last administered dose of ARN 509. |
| Study Visits and Procedures |
|  |
| Screening Visits and Procedures |
| All potential subjects are referred from their primary urologists. They will be screened by a member of the study team, and the procedure will include:   1. a detailed history and clinical examination 2. screening investigations including FBC, renal panel. liver panel, thyroid function, urinalysis, PSA, hepatitis screen, chest X ray and ECG   These screening tests should be done after clinical eligibility is ascertained and the recruitment into the trial should occur within 4 weeks of screening.  In addition to the blood samples taken for screening, peripheral blood will be collected at the following occasions: (i) during trial entry, at the completion of ARN 509 therapy timed together with pre-operative blood tests, and after surgery timed together with post surgery PSA assessment About 50ml of blood will be taken at each venepuncture. A total of 175 ml for all study related blood tests is expected.  Voided urine about 150ml will be taken at the same timing as peripheral blood. A total of 470ml of urine will be expected to be collected.  These samples may be tested for the following, inclusive but not limited to proteomics, genetics and cytokines testing.  The samples will be retained at the end of the study by the investigator and stored in a secure room in SGH. Only the study team will have access to the study samples. In the event the samples are required to be transported outside SGH to a service provider, these samples will be coded.  The subject can withdraw consent to participate in the sample collection and/or for their samples to be retained at any time and still continue with the study.  Sample analysis is restricted to purposes of this study and not for future unknown usage. |
| Study Visits and Procedures |
| The following are scheduled during the intervention phase of the study:   1. week 0 where patients will receive ARN 509 2. week 4 after commencement of ARN 509 for clinical consult, physical examination and QoL outcomes 3. week 12 at completion of ARN 509 treatment, where clinical examination, blood investigations, QoL outcomes and adverse effects monitoring is performed 4. Standard of care surgery by week 16 5. Final visit at weeks 20-24 for a clinical examination, QoL outcomes, adverse effects monitoring and serum PSA assessment |
| Final Study Visit: |
| The final study visit is about 4-6 weeks after standard of care surgery, where clinical examination, QoL outcomes measurement, adverse effects monitoring and serum PSA will be ascertained. |
| Post Study Follow up and Procedures |
| A post study follow-up is planned only for individuals who have drug related adverse effects that are present at the initial study visit. These patients will be monitored until resolution of adverse effects. |
| **TRIAL MATERIALS** |
|  |
| Trial Product (s) |
| This information is provided in section 4 (Effects in Humans) of the Investigator’s brochure which is attached.  Apalutamide is not marketed in Singapore, and the use of the study drug will be followed as per protocol. |
| Storage and Drug Accountability |
| The product comes as 60mg tablet stored in high density polyethylene (HDPE) bottles with child-resistant closures (CRC) and tamper-proof heat induction seals.  They are to be stored at room temperature in lock and key. |
|  |
| **TREATMENT** |
| Rationale for Selection of Dose |
| Preceding phase I and II studies have determined a daily dose of 240mg is efficacious and safe ( See Investigator Brochure Section 4.2 and Appendix B.1) |
| Study Drug Formulations |
| They will be dispensed as tablets to be stored at room temperature. |
| Study Drug Administration |
| The study drug should be taken once in the morning with or without food. |
| Specific Restrictions / Requirements |
| The following drugs should be stopped during the study period when ARN 509 is being consumed:   - Aminophylline/theophylline -  Atypical antipsychotics (eg, clozapine, olanzapine, risperidone, ziprasidone) -  Buproprion -  Lithium -  Meperidine and pethidine -  Phenothiazine antipsychotics (eg, chlorpromazine, mesoridazine, thioridazine) -  Tricyclic and tetracyclic antidepressants (eg, amitriptyline, desipramine, doxepin,   imipramine, maprotiline, mirtazapine) |
| Blinding |
| Not applicable. |
| Concomitant therapy |
| All medications (prescription and over the counter), vitamin and mineral supplements, and / or herbs taken by the participant should be documented. |
|  |
| **SAFETY MEASUREMENTS** |
| Definitions |
| An adverse event (AE) is any untoward medical occurrence in a patient or clinical investigation subject administered a pharmaceutical product and which does not necessarily have a causal relationship with this treatment.  A serious adverse event (SAE) or reaction is any untoward medical occurrence that at any dose:   - results in death - is life-threatening - requires inpatient hospitalisation or prolongation of existing hospitalisation - results in persistent or significant disability/incapacity or - is a congenital anomaly/birth defect - is a medical event that may jeopardize the patient and may require medical or surgical intervention to prevent one of the outcomes listed above.   Potential Discomforts, Side Effects, and Risks Associated with Apalutamide  The possible discomforts, side effects, and risks related to apalutamide treatment are not all known. Though, most side effects are not serious, some may be serious and may require treatment or additional testing. This section describes how frequently side effects occurred in subjects who were treated with apalutamide.  There may be risks with the use of apalutamide that are not yet known. You will be watched carefully during this study for any side effects. Sometimes during a study the sponsor or study doctor may learn new facts about the study medications. It is possible that this information might make you change your mind about being in the study. If new information is discovered, your study doctor will tell you about it.  Side effects may be mild or serious. All medicines have the potential to cause an allergic reaction. Some allergic reactions and side-effects may potentially be life threatening. If you do experience any side effects, your doctor may need to give you medicines to help lessen the side effect. Many side effects may go away soon after you stop taking the study medicine. In some cases, side effects can be serious or long lasting.  If you experience a side effect, your treating doctor may change your study drug doses in an effort to decrease or stop any side effects. If severe side effects do develop, you and your doctor may decide that it is in your best interest to stop taking part in the study. If you choose, you always have the right to withdraw from the study. In addition, you will be provided with the telephone numbers for people who can answer any questions about the study, your rights as a study participant and for you to report any side effects.  Risks and side effects that may be possibly related to apalutamide include:   | **Very Common (>10%)** | **Common (1-10%)** | **Very Rare ( < 1%)** | | --- | --- | --- | | Fatigue | Itching | Seizures | | Diarrhoea | Falls |  | | Nausea | Insomnia |  | | Vomiting | Increase in blood cholesterol |  | | Skin rash | Increase in blood triglycerides |  | | Abdominal pain |  |  | | Changes in thyroid function |  |  | | Taste alterations |  |  | | Constipation |  |  | | Decreased appetite |  |  | | Hot flashes |  |  | | Dizziness |  |  |   Seizures have been observed very rarely in patients taking part in apalutamide studies that are still blinded (blinded means that neither the doctor nor the patient knows whether the patient is receiving apalutamide or placebo). Your doctor will confirm that you have no history of seizures and will check throughout the study that you are not taking other medications that can increase your risk of seizures. Please inform your doctor of all medications you take and any changes in medications. If you think you might have had a seizure, or convulsion, or have lost consciousness (passed out), let your doctor know right away.  Some patients on apalutamide studies have developed a rash. The cause of the rash is not known. The rash may be confined to one area of your body or may involve your entire body. If you start to develop a rash on any part of your body, or if you develop any other symptoms that are worrisome to you, contact your study doctor right away.  The effect of the study drug on your semen is unknown. To avoid risk of drug exposure to your partner through the semen (even in men with vasectomies [tubes that carry semen from the testicles have been cut]), or cause harm to an unborn child, you must use a condom during sexual activity while on study drug and for 3 months following the last dose of study drug.  Apalutamide may cause harm to the unborn child. From when you start taking the study drug until 3 months after your last dose of study drug, you must use a condom and another effective method of birth control when you have sex with a woman of child-bearing potential. The type of birth control you use must be discussed with, and approved by, the study doctor before you begin the study. This is done to prevent pregnancy. If your partner becomes pregnant in the time between when you start taking the study drug until 3 months after your last dose of drug, you must tell the study doctor immediately.  Donation of sperm is not allowed during the study and for 3 months following the last dose of study drug.  You should advise your study doctor if you father a child while participating in the research project. The doctor will advise you on any appropriate medical attention for your partner should this be necessary. The sponsor may ask you and your partner to allow him/her to collect information about her pregnancy and the health of the baby. |
| Collecting, Recording and Reporting of Adverse Events and Serious Adverse Events to CIRB |
| Reporting of adverse events involves the PI submitting to the approving CIRB the completed SAE Reporting Form within the stipulated timeframe. PI is responsible for informing the institution representative (local SAE resulting in death), sponsor or regulatory bodies as required and appropriate.  Reporting timeline to CIRB:   - SAE that result in death, regardless of causality, should be reported immediately - within 24 hours of the PI becoming aware of the event. - Local life-threatening (unexpected/ expected) SAE should be reported no later than 7 calendar days after the Investigator is aware of the event, followed by a complete report within 8 additional calendar days. - Local unexpected SAE that are related events, but not life-threatening, should be reported no later than 15 calendar days after the investigator is aware of the event. - An increase in the rate of occurrence of local expected SAE, which is judged to be clinically important, should be reported within 15 calendar days after the PI is aware of the event. - Local expected SAE should be reported annually (together with Study Status Report for annual review). - Local unexpected and unlikely related SAE that are not life-threatening should also be reported annually (together with Study Status Report for annual review). - Local unexpected AE that are related events should be reported at least annually (together with Study Status Report for annual review). - Non-local unexpected SAE that are fatal or life threatening and definitely/probably/possibly related should be reported not later than 30 calendar days after the PI is aware of the event. |
| Collecting, Recording and Reporting of Serious Adverse Events (SAEs) to the Health Science Authority (HSA) |
| All SAEs that are unexpected and related to the study drug will be reported to HSA. All SAEs will be reported to HSA according to the HSA Guidance for Industry “Safety Reporting Requirements for Clinical Drug Trials.”  The investigator is responsible for informing HSA no later than 15 calendar days after first knowledge that the case qualifies for expedited reporting. Follow-up information will be actively sought and submitted as it becomes available. For fatal or life-threatening cases, HSA will be notified as soon as possible but no later than 7 calendar days after first knowledge that a case qualifies, followed by a complete report within 8 additional calendar days. Safety Reporting Requirement for Johnson & Johnson & Johnson All AEs will be recorded from the time the patient signs informed consent to 28 days after the last dose of the study drug(s). AEs and SAEs will be recorded on the AE page of the CRF and in the patient’s source documents. All SAEs must be reported to Johnson & Johnson within 24 hours of the study site staff’s knowledge of the event, using the Johnson & Johnson SAE Report Form. |
| - - 1. **Special Reporting Situations**   Safety events of interest for apalutamide that requires reporting and/or safety evaluation include, but are not limited to:   - Apalutamide exposure during pregnancy (paternal) - Overdose of Apalutamide - Exposure to Apalutamide from breastfeeding - Suspected abuse/misuse of Apalutamide - Inadvertent or accidental exposure to Apalutamide - Any failure of expected pharmacological action (ie. Lack of effect) of Apalutamide - Medication error involving Apalutamide (with or without patient exposure to Apalutamide, e.g. name confusion - Suspected transmission of any infectious agent via administration of Apalutamide - Unexpected therapeutic or clinical benefit from use of Apalutamide   These safety events may not meet the definition of an adverse event; however, from Johnson & Johnson’s perspective, they are treated in the same manner as adverse events.  Any special situation that meets the criteria of a serious adverse event should be recorded on Johnson & Johnson Serious Adverse Event Report Form and be reported to Johnson & Johnson within 24 hours of becoming aware of the event.   - - 1. **Pregnancy**   If a female partner of a male patient taking the Study Product becomes pregnant, the male patient taking Study Product should notify the Investigator, and the pregnant female partner should be advised to call their healthcare provider immediately. The pregnancy, suspected pregnancy or positive pregnancy test must be reported to Johnson & Johnson within 24 hours of awareness of the pregnancy, using the Johnson & Johnson Pregnancy Notification Form.   - - 1. **Product Quality Complaints (PQCs)**   A PQC may have an impact on the safety and efficacy of the product. Timely, accurate, and complete reporting and analysis of PQC information from studies are crucial for the protection of patients, investigators, and Johnson & Johnson, and are mandated by regulatory agencies worldwide. Johnson & Johnson has established procedures in conformity with regulatory requirements worldwide to ensure appropriate reporting of PQC information. Lot and/or Batch #s shall be collected for any reports failure of expected pharmacological action (i.e., lack of effect).  All initial PQCs involving apalutamib must be reported to Johnson & Johnson by the Investigators within 24 hours after being made aware of the event.  If the defect for apalutamide is combined with a serious adverse event, the Investigators must report the PQC to Johnson & Johnson according to the serious adverse event reporting timelines. Safety Monitoring Plan |
| The study team members will perform the safety monitoring in this study.  Upon recruitment, all patients will be given the contact number of the PI which is accessible 24 hours. They will be instructed to report all suspected adverse effects within 24 hours of occurrence to the PI.  The scheduled collection of safety data will occur at weeks 6 and 12 and 20-24 after initiation of ARN 509, where a study team member will enquire about possible adverse effects experienced by the subjects.  A 6 monthly review of the safety data and of the collected study data will be performed by the study team.  Please include details on the Data Safety Monitoring Plan (DSMP) for the research study. Please discuss the plans in place to ensure the safety and well being of subjects, and integrity of data collected. |
| Complaint Handling |
| All complaints will be directed towards the Director, Division of Research, Singapore General Hospital.  The complaints will be investigated in conjunction with inputs from the Principal Investigator.  The details of all complaints will be recorded and kept confidential. |
|  |
| **DATA ANALYSIS** |
| Data Quality Assurance |
| The source data will be the case records and all data entries will be cross-checked by the PI after data entry and prior to data analysis. |
| Data Entry and Storage |
| Data will be kept encrypted and electronically within a SGH network computer secured with a user password and room secured with card access. |
|  |
| **SAMPLE SIZE AND STATISTICAL METHODS** |
| Determination of Sample Size |
| There is historical study data showing no more than pathological down staging of 15% in neoadjuvant studies with conventional androgen deprivation.  Phase II studies in advanced prostate cancer patients show a 89% reduction of PSA levels of 50% or more in patients previously treated with conventional androgen deprivation.  For this study with treatment naïve patients, we extrapolate that the study drug will exert a conservative effect equivalent to 40% of patients with significant tissue response with low residual disease burden.  Using a one sided P value of 0.05, beta of 0.2, a single arm study of about n=30 patients will be required. |
| Statistical and Analytical Plans |
| - - - 1. General Considerations   As this is a single arm study with no control group, all analysis will be performed with the intent of characterising outcomes in this group, so as to form the background for future related interventional studies. |
| - - - 1. Safety Analyses   Drug related adverse effects will be graded according the CTCAE grading scale. All AEs reported will be graded according to scale and the prevalence calculated. |
| - - - 1. Interim Analyses   An interim analysis of outcomes will be performed when 50% accrual is completed.   - - - 1. Describe the types of statistical interim analyses, including their timing.   This will be performed when 50% accrual is completed, and will include:   1. safety outcomes analysis 2. study primary and secondary objectives as listed |
| **DIRECT ACCESS TO SOURCE DATA/DOCUMENTS** The investigator(s)/institution(s) will permit study-related monitoring, audits and/or IRB review and regulatory inspection(s), providing direct access to source data/document. **QUALITY CONTROL AND QUALITY ASSURANCE** The collected data will be compared with the source data ( patient case records). This will be cross checked by at least 2 members of the study team. The access to the data will also be maintained under lock and key and only accessible to the PI and co-PIs.  Evaluation of data quality is every 6 months.  The PI and co-investigators would perform the data and safety monitoring. **ETHICAL CONSIDERATIONS** This study will be conducted in accordance with the ethical principles that have their origin in the Declaration of Helsinki and that are consistent with the Singapore Good Clinical Practice and the applicable regulatory requirements.  This final study protocol, including the final version of the Patient Information and Informed Consent Form, must be approved in writing by the Centralised Institutional Review Board (CIRB) and regulatory approval from Health Sciences Authority (HSA), prior to enrolment of any patient into the study.  The principle investigator is responsible for informing the CIRB and HSA of any amendments to the protocol or other study-related documents, as per local requirement. |
| Informed Consent |
| The consent will be obtained after the patient is aware of his diagnosis and has consented to undergoing radical prostatectomy for prostate cancer.  Consent will be obtained by PI and co-PIs, and will be taken in the outpatient clinic. This is the most common location where patients are told of their diagnosis and counselled for surgery.  Patients will be told that their treatment and follow-up will not be influenced by their decision on enrolment into the study.  We will recruit English speaking patients. There will be no minors. |
| Confidentiality of Data and Patient Records |
| Research data will be stored on a network protected computer secured within a room in the department of Urology accessible only with a security pass.  Research data that is sent to external parties will be coded and de-identified. |
|  |
| **PUBLICATIONS** |
| The publications will be authored by the study team members and will reflect and be identified as from Singapore General Hospital, with acknowledgements of funding from Johnson and Johnson. |
|  |
| **RETENTION OF TRIAL DOCUMENTS** |
| Records for all participants, including CRFs, all source documentation (containing evidence to study eligibility, history and physical findings, laboratory data, results of consultations, etc.) as well as IRB records and other regulatory documentation should be retained by the PI in a secure storage facility. The records should be accessible for inspection and copying by authorized authorities.  The records will be kept under lock and key in a room accessible only to study PI and co-PIs. They will be retained for 5 years after completion of the study. |

| **FUNDING and INSURANCE** |
| --- |
| Johnson and Johnson will provide SGD 50K for trial related expenses, and the study drug in kind.  Indemnity insurance will be provided by Singapore General Hospital. |

**References**

1. D'Amico AV, Whittington R, Malkowicz SB, Schultz D, Blank K, Broderick GA, et al. Biochemical outcome after radical prostatectomy, external beam radiation therapy, or interstitial radiation therapy for clinically localized prostate cancer. JAMA 1998;280(11):969-74.

2. Bolla M, Collette L, Blank L, Warde P, Dubois JB, Mirimanoff RO, et al. Long-term results with immediate androgen suppression and external irradiation in patients with locally advanced prostate cancer (an EORTC study): a phase III randomised trial. Lancet 2002;360(9327):103-6.

3. Messing EM, Manola J, Yao J, Kiernan M, Crawford D, Wilding G, et al. Immediate versus deferred androgen deprivation treatment in patients with node-positive prostate cancer after radical prostatectomy and pelvic lymphadenectomy. Lancet Oncol 2006;7(6):472-9.

4. Alvin LW, Gee SH, Hong HH, Christopher CW, Henry HS, Weber LK, et al. Oncological outcomes following robotic-assisted radical prostatectomy in a multiracial Asian population. J Robot Surg 2015;9(3):201-9

5. Beer TM, Garzotto M, Lowe BA, Ellis WJ, Montalto MA, Lange PH, et al. Phase I Study of Weekly Mitoxantrone and Docetaxel before Prostatectomy in Patients with High-Risk Localized Prostate Cancer. Clinical Cancer Research 2004;10(4):1306-1311.

6. Clark PE, Peereboom DM, Dreicer R, Levin HS, Clark SB, Klein EA. Phase II trial of neoadjuvant estramustine and etoposide plus radical prostatectomy for locally advanced prostate cancer. Urology 2001;57(2):281-285.

7. Dreicer R, Magi-Galluzzi C, Zhou M, Rothaermel J, Reuther A, Ulchaker J, et al. Phase II trial of neoadjuvant docetaxel before radical prostatectomy for locally advanced prostate cancer. Urology 2004;63(6):1138-1142.

8. Febbo PG, Richie JP, George DJ, Loda M, Manola J, Shankar S, et al. Neoadjuvant Docetaxel before Radical Prostatectomy in Patients with High-Risk Localized Prostate Cancer. Clinical Cancer Research 2005;11(14):5233-5240.

9. Konety BR, Eastham JA, Reuter VE, Scardino PT, Donat SM, Dalbagni G, et al. Feasibility of Radical Prostatectomy After Neoadjuvant Chemohormonal Therapy for Patients With High Risk or Locally Advanced Prostate Cancer: Results of a Phase I/II Study. The Journal of Urology 2004;171(2, Part 1):709-713.

10. Berglund RK, Tangen CM, Powell IJ, Lowe BA, Haas GP, Carroll PR, et al. Ten-year follow-up of neoadjuvant therapy with goserelin acetate and flutamide before radical prostatectomy for clinical T3 and T4 prostate cancer: update on Southwest Oncology Group Study 9109. Urology 2012;79(3):633-7.

11. Gleave ME, Goldenberg SL, Chin JL, Warner J, Saad F, Klotz LH, et al. RANDOMIZED COMPARATIVE STUDY OF 3 VERSUS 8-MONTH NEOADJUVANT HORMONAL THERAPY BEFORE RADICAL PROSTATECTOMY: BIOCHEMICAL AND PATHOLOGICAL EFFECTS. The Journal of Urology;166(2):500-507.

12. Labrie F, Cusan L, Gomez J-L, Diamond P, Suburu R, Lemay M, et al. Down-staging of early stage prostate cancer before radical prostatectomy: The first randomized trial of neoadjuvant combination therapy with flutamide and a luteinizing hormone-releasing hormone agonist. Urology 1994;44(6):29-37.

13. Klotz L, O'Callaghan C, Ding K, Toren P, Dearnaley D, Higano CS, et al. Nadir testosterone within first year of androgen-deprivation therapy (ADT) predicts for time to castration-resistant progression: a secondary analysis of the PR-7 trial of intermittent versus continuous ADT. J Clin Oncol 2015;33(10):1151-6.

14. Taplin M-E, Montgomery B, Logothetis CJ, Bubley GJ, Richie JP, Dalkin BL, et al. Intense Androgen-Deprivation Therapy With Abiraterone Acetate Plus Leuprolide Acetate in Patients With Localized High-Risk Prostate Cancer: Results of a Randomized Phase II Neoadjuvant Study. Journal of Clinical Oncology 2014;32(33):3705-3715.

15. Ishizaki F, Nishiyama T, Kawasaki T, Miyashiro Y, Hara N, Takizawa I, et al. Androgen deprivation promotes intratumoral synthesis of dihydrotestosterone from androgen metabolites in prostate cancer. Sci Rep 2013;3:1528.

16. Hobisch A, Hoffmann J, Lambrinidis L, Eder IE, Bartsch G, Klocker H, et al. Antagonist/agonist balance of the nonsteroidal antiandrogen bicalutamide (Casodex) in a new prostate cancer model. Urol Int 2000;65(2):73-9.

17. Culig Z, Hoffmann J, Erdel M, Eder IE, Hobisch A, Hittmair A, et al. Switch from antagonist to agonist of the androgen receptor blocker bicalutamide is associated with prostate tumour progression in a new model system. British Journal of Cancer 1999;81(2):242-251.

18. Clegg NJ, Wongvipat J, Joseph JD, Tran C, Ouk S, Dilhas A, et al. ARN-509: A Novel Antiandrogen for Prostate Cancer Treatment. Cancer Research 2012;72(6):1494-1503.

19. Smith MR, Antonarakis ES, Ryan CJ, Berry WR, Shore ND, Liu G, et al. Phase 2 Study of the Safety and Antitumor Activity of Apalutamide (ARN-509), a Potent Androgen Receptor Antagonist, in the High-risk Nonmetastatic Castration-resistant Prostate Cancer Cohort. European Urology 2016;70(6):963-970.

20. Bossi A, Dearnaley D, McKenzie M, Baskin-Bey E, Tyler R, Tombal B, et al. ATLAS: A phase 3 trial evaluating the efficacy of apalutamide (ARN-509) in patients with high-risk localized or locally advanced prostate cancer receiving primary radiation therapy. Annals of Oncology 2016;27(suppl_6):769TiP-769TiP.

21. Chi KN, Chowdhury S, Radziszewski P, Lebret T, Ozguroglu M, Sternberg C, et al. TITAN: A randomized, double-blind, placebo-controlled, phase 3 trial of apalutamide (ARN-509) plus androgen deprivation therapy (ADT) in metastatic hormone-sensitive prostate cancer (mHSPC). Annals of Oncology 2016;27(suppl_6):771TiP-771TiP.

22. Abstract COP-5. BJU International 2014;113:1-37.

23. Pound CR, Partin AW, Eisenberger MA, Chan DW, Pearson JD, Walsh PC. Natural history of progression after PSA elevation following radical prostatectomy. Jama 1999;281(17):1591-1597.

24. Stephenson AJ, Scardino PT, Kattan MW, Pisansky TM, Slawin KM, Klein EA, et al. Predicting the outcome of salvage radiation therapy for recurrent prostate cancer after radical prostatectomy. Journal of Clinical Oncology 2007;25(15):2035-2041.

25. Han M, Partin AW, Pound CR, Epstein JI, Walsh PC. LONG-TERM BIOCHEMICAL DISEASE-FREE AND CANCER-SPECIFIC SURVIVAL FOLLOWING ANATOMIC RADICAL RETROPUBIC PROSTATECTOMY: The 15-Year Johns Hopkins Experience. Urologic Clinics of North America 2001;28(3):555-565.

26. Liesenfeld L, Kron M, Gschwend JE, Herkommer K. Prognostic Factors for Biochemical Recurrence More than 10 Years after Radical Prostatectomy. The Journal of Urology;197(1):143-148.

27. Pietzak EJ, Eastham JA. Neoadjuvant Treatment of High-Risk, Clinically Localized Prostate Cancer Prior to Radical Prostatectomy. Current Urology Reports 2016;17(5):37.

28.       Symmans WF, Peintinger F, Hatzis C, Rajan R, Kuerer H, Valero V, et al. Measurement of Residual Breast Cancer Burden to Predict Survival After Neoadjuvant Chemotherapy. Journal of Clinical Oncology 2007;25(28):4414-4422.

29.       Efstathiou E, Abrahams NA, Tibbs RF, Wang X, Pettaway CA, Pisters LL, et al. Morphologic characterization of preoperatively treated prostate cancer: toward a post-therapy histologic classification. Eur Urol 2010;57(6):1030-8.

30.       Murphy C, True L, Vakar-Lopez F, Xia J, Gulati R, Montgomery B, et al. A Novel System for Estimating Residual Disease and Pathologic Response to Neoadjuvant Treatment of Prostate Cancer. Prostate 2016;76(14):1285-92

**List of Attachments**

| Appendix 1 | Study Schedule |
| --- | --- |
| Appendix 2  Table 1 | Blood Sampling Summary  Trial Flow chart |
|  |  |
| **Appendix 1 Study schedule**  Total n=30: Obtain informed consent.  Screen potential participants by inclusion and exclusion criteria.  Physical examination, ECG, Chest X-ray and screening investigations  QoL outcomes assessment  Visit 1  Prior to  Enrolment  Entry into trial  Blood and urine samples collected for translational research  Commence ARN 509 administration  Visit 2  Week 0  Follow-up assessments for drug related side effects  Physical examination  QoL outcomes assessment  Visit 3  Week 4  Follow-up assessments  (physical examination, blood tests (FBC, renal panel, urine tests, PSA levels), urinalysis and QoL outcomes)  (blood and urine samples for translational research)  Visit 4  Week 12  Standard of care surgery  Week 12-16  Visit 5  Week 20-24 | **Final Assessments**  Physical examination  Blood tests (PSA level)  Blood and urine samples for translational research  QoL outcomes |
|  |  |

**Appendix 2 Blood sampling schedule**

| Patients on treatment arm with ARN509 | | | | | | | | |
| --- | --- | --- | --- | --- | --- | --- | --- | --- |
| Trial Period | | Screening | ARN 509 | | Follow-up (1) | Standard of care surgery | Follow-up (2) | Remarks |
| Week | | -4 | 0 | 4 | 12 | 12-16 | 20-24 |  |
|  | |  |  |  |  |  |  |  |
| **Laboratory procedures** | | | | | | | | |
| FBC with differentials | | X |  |  | x |  |  |  |
| Renal panel including calcium and phosphate levels | | x |  |  | x |  |  |  |
| Liver panel | | X |  |  | x |  |  |  |
| PSA levels | | X |  |  | x |  | x |  |
| Urinalysis | | X |  |  | x |  |  |  |
| Thyroid function test | | X |  |  |  |  |  |  |
| Hepatitis and HIV status | | X |  |  |  |  |  |  |
|  | | | | | | | | |
| Trial Period | | Screening | ARN 509 | | Follow-up (1) | Standard of care surgery | Follow-up (2) | Remarks |
| Week | | -4 | 0 | 4 | 12 | 12-16 | 20-24 |  |
| **Protocol specific collection** | | | | | | | | |
| Blood | CTCs from peripheral blood |  | x |  | x |  | x |  |
|  | Plasma ( ctDNA, miRNA, exosomes) |  | X |  | x |  | x |  |
|  | PBMC genotypic analysis |  | X |  | x |  | x |  |
|  | Serum (biomarkers) |  | X |  | x |  | x |  |

**Table 1. ARN 509 Phase II Investigator initiated trial Singapore General Hospital - Trial Flow Chart**

| Patients on treatment arm with ARN509 | | | | | | | |
| --- | --- | --- | --- | --- | --- | --- | --- |
| Trial Period | Screening | ARN 509 | | Follow-up (1) | Standard of care surgery | Follow-up (2) | Remarks |
| Week | -4 | 0 | 4 | 12 | 12-16 | 20-24 |  |
|  |  |  |  |  |  |  |  |
| **Administrative procedures** | | | | | | | |
| Informed consent for trial and biochemical research | X |  |  |  |  |  |  |
| Inclusion/ exclusion criteria | x |  |  |  |  |  |  |
| Subject identification card | x |  |  |  |  |  |  |
| Medical history |  |  |  |  |  |  |  |
| ECOG performance assessment | x |  |  |  |  |  |  |
| Concomitant medication review | x |  |  |  |  |  |  |
|  |  |  |  |  |  |  |  |
| **Clinic procedures** | | | | | | | |
| Full physical examination | X |  |  | X | x | x |  |
| Vital signs, Body mass index | x |  |  | X | x | x |  |
| 12 lead ECG | x |  |  |  |  |  |  |
| Chest X Ray | x |  |  |  |  |  |  |
| ARN509 administration |  | X | X | x |  |  |  |
| SOC surgery |  |  |  |  | x |  |  |
| Adverse Events monitoring |  | x | x | X | x | x |  |
| Assessment of surgical complications |  |  |  |  | x | x |  |
| Assessment of QoL outcomes (QLQC30, PR25 and SHIM) | x |  | x | x |  | x |  |
|  |  |  |  |  |  |  |  |
| **Laboratory procedures** | | | | | | | |
| FBC with differentials | X |  |  | x |  |  |  |
| Renal panel including calcium and phosphate levels | x |  |  | x |  |  |  |
| Liver panel | X |  |  | x |  |  |  |
| PSA levels | X |  |  | x |  | x |  |
| Urinalysis | X |  |  | x |  |  |  |
| Thyroid function test | X |  |  |  |  |  |  |
| Hepatitis and HIV status | X |  |  |  |  |  |  |

| Trial Period | | Screening | ARN 509 | | Follow-up (1) | Standard of care surgery | Follow-up (2) | Remarks |
| --- | --- | --- | --- | --- | --- | --- | --- | --- |
| Week | | -4 | 0 | 4 | 12 | 12-16 | 20-24 |  |
| **Protocol specific collection** | | | | | | | | |
| Blood | CTCs from peripheral blood |  | x |  | x |  | x |  |
|  | Plasma ( ctDNA, miRNA, exosomes) |  | X |  | x |  | x |  |
|  | PBMC genotypic analysis |  | X |  | x |  | x |  |
|  | Serum (biomarkers) |  | X |  | x |  | x |  |
| Urine | Urine (miRNA, exosomes) |  | X |  | x |  | x |  |
| Tissue | Pre-treatment prostate cancer tissue (needle biopsy)  (DNA, RNA, IHC, methylation status) |  | x |  |  |  |  |  |
|  | Post -treatment prostate cancer tissue (whole mount prostate tissue)  (DNA, RNA, IHC, methylation status) |  |  |  |  | x |  |  |
|  | FFPE benign tissue via laser capture microdissection (DNA, RNA, IHC) |  | x |  |  | x |  |  |
